# Comprehensive Analysis of Expression and Prognostic Value of MS4As in Glioma

**DOI:** 10.3389/fgene.2022.795844

**Published:** 2022-06-06

**Authors:** Yingying Zeng, Peixin Tan, Chen Ren, Lianxuan Gao, Yulei Chen, Shushu Hu, Nan Tang, Chen Chen, Shasha Du

**Affiliations:** ^1^ Department of Radiation Oncology, Nanfang Hospital, Southern Medical University, Guangzhou, China; ^2^ Department of Radiology, Guangdong Provincial People’s Hospital, Guangdong Academy of Medical Sciences, Guangzhou, China

**Keywords:** MS4A, glioma, biomarker, prognosis, immune infiltration

## Abstract

Glioma is the most common malignancy of the nervous system with high mortality rates. The MS4A family members have been reported as potential prognostic biomarkers in several cancers; however, the relationship between the MS4A family and glioma has not been clearly confirmed. In our study, we explored the prognostic value of MS4As as well as their potential pro-cancer mechanisms of glioma. Using bioinformatics analysis methods based on the data from public databases, we found that the expression of MS4A4A, MS4A4E, MS4A6A, MS4A7, TMEM176A, and TMEM176B was significantly overexpressed in glioma tissues compared with that of normal tissues. The Kaplan–Meier method and Cox proportional hazards models revealed that high levels of MS4As can be associated with a poorer prognosis; TMEM176A, TMEM176B, age, WHO grade, and IDH status were identified as independent prognostic factors. Enrichment analysis predicted that MS4As were related to tumor-related pathways and immune response, which might regulate the process of MS4As promoting tumorigenesis. Additionally, we analyzed the correlations of MS4A expression with immune cells and immune inhibitory molecules. Finally, data from the cell culture suggested that knockdown of the TMEM176B gene contributes to the decreased proliferation and migration of glioma cells. In conclusion, MS4A4A, MS4A4E, MS4A6A, MS4A7, TMEM176A, and TMEM176B may act as potential diagnostic or prognostic biomarkers in glioma and play a role in forming the immune microenvironment in gliomas.

## Introduction

Glioma is the most common malignant tumor in the central nervous system (Ostrom et al., 2014) and also the leading cause of cancer-related deaths associated with the central nervous system (Siegel et al., 2016). According to histopathological and clinical criteria, it can be classified into four grades (Grade I–IV) (Louis et al., 2016). Despite advances in treatment strategies for glioma over the past decades, the median overall survival after diagnosis is still approximately 15 months, and long-term survival is unsatisfactory (Wen et al., 2020). Due to the poor prognosis of glioma patients, it is urgent to discover new biomarkers or potential molecular targets in order to improve diagnosis, prognosis, and treatment of glioma.

The membrane-spanning 4A (MS4A) family belongs to a cluster of structurally related proteins with four transmembrane-spanning domains, and it has 18 members: *MS4A1*, *MS4A2*, *MS4A3*, *MS4A4A*, *MS4A4E*, *MS4A5*, *MS4A6A*, *MS4A6E*, *MS4A7*, *MS4A8B*, *MS4A10*, *MS4A12*, *MS4A13*, *MS4A14*, *MS4A15*, *MS4A18*, *TMEM176A,* and *TMEM176B* (Mattiola et al., 2021). *MS4A1* (CD20), *MS4A2* (FcεRIβ), *MS4A3* (HTm4), and *MS4A4A* play important roles in immunity, whereas the expression and function of other members of the family are not exact (Silva-Gomes et al., 2021). However, evidence from pre-clinical models and genetic evidence from humans suggest that members of the MS4A family have key roles in different pathological settings, including cancer, allergies, and metabolic, neurodegenerative, and autoimmune diseases (Mattiola et al., 2021). Overexpression of the MS4A family can be seen in several cancers, and they are closely related to the clinicopathological characteristics and prognosis of several tumors, such as gastric cancer (Sun et al., 2018), colon cancer (He et al., 2017), breast cancer, melanoma, lymphoma, and hepatocellular carcinoma (Cuajungco et al., 2012). However, the correlations between MS4A expression and prognosis in glioma remain unclear.

In this study, we first investigated the expression patterns of MS4A family genes in the glioma tissues and explored the potential correlation between the expression level of MS4A family genes and the clinical prognosis of glioma cases within TCGA databases. The potential biological functions and signal pathways which MS4As participate in glioma were analyzed. Moreover, TIMER2.0 was used to assess the effect of MS4As on immune cell infiltration and their correlation with immune cell gene marker expression in glioma. Finally, we screened out TMEM176B, which is overexpressed in glioma and significantly correlated with the prognosis of glioma patients. To further analyze the cellular function of TMEM176B, we have successfully established the TMEM176B-knockdown glioma cell lines and explored the effect of TMEM176B on cell function. Our study shows that the MS4A family members may be potential therapeutic targets with a promising prognostic value in glioma patients.

## Materials and Methods

### Public Databases

RNA-seq data of GBM (glioblastoma) (*n* = 174), LGG (low-grade glioma) (*n* = 529), and normal brain samples were downloaded from TCGA and GTEx using UCSC Xena (https://xenabrowser.net/datapages/) (Carter et al., 2010). These data were uniformly transformed into TPM (transcripts per million reads) by the Toil process for comparative analyses. Table 1 shows the details of 703 glioma patients.

**TABLE 1:** Baseline data from TCGA-GBM and TCGA-LGG.

Characteristic	MS4A4A			MS4A4E			MS4A6A			MS4A7			TMEM176A			TMEM176B		
	Low expression	High expression	*p*	Low expression	High expression	*p*	Low expression	High expression	*p*	Low expression	High expression	*p*	Low expression	High expression	*p*	Low expression	High expression	*p*
*n*	348	348		348	348		348	348		348	348		348	348		348	348	
Gender, *n* (%)			<0.001			0.007			0.078			0.592			0.078			0.146
Female	157 (22.6%)	141 (20.3%)		167 (24%)	131 (18.8%)		161 (23.1%)	137 (19.7%)		153 (22%)	145 (20.8%)		161 (23.1%)	137 (19.7%)		159 (22.8%)	139 (20%)	
Male	191 (27.4%)	207 (29.7%)		181 (26%)	217 (31.2%)		187 (26.9%)	211 (30.3%)		195 (28%)	203 (29.2%)		187 (26.9%)	211 (30.3%)		189 (27.2%)	209 (30%)	
Age, *n* (%)						<0.001			<0.001			0.189			<0.001			<0.001
≤60	296 (42.5%)	257 (36.9%)	<0.001	302 (43.4%)	251 (36.1%)		307 (44.1%)	246 (35.3%)		284 (40.8%)	269 (38.6%)		305 (43.8%)	248 (35.6%)		305 (43.8%)	248 (35.6%)	
>60	52 (7.5%)	91 (13.1%)		46 (6.6%)	97 (13.9%)		41 (5.9%)	102 (14.7%)		64 (9.2%)	79 (11.4%)		43 (6.2%)	100 (14.4%)		43 (6.2%)	100 (14.4%)	
WHO grade, *n* (%)						<0.001			<0.001			<0.001			<0.001			<0.001
G2	146 (23%)	78 (12.3%)	<0.001	170 (26.8%)	54 (8.5%)		165 (26%)	59 (9.3%)		144 (22.7%)	80 (12.6%)		142 (22.4%)	82 (12.9%)		150 (23.6%)	74 (11.7%)	
G3	140 (22%)	103 (16.2%)		117 (18.4%)	126 (19.8%)		131 (20.6%)	112 (17.6%)		130 (20.5%)	113 (17.8%)		143 (22.5%)	100 (15.7%)		139 (21.9%)	104 (16.4%)	
G4	27 (4.3%)	141 (22.2%)		28 (4.4%)	140 (22%)		12 (1.9%)	156 (24.6%)		44 (6.9%)	124 (19.5%)		24 (3.8%)	144 (22.7%)		19 (3%)	149 (23.5%)	
IDH status, *n* (%)			<0.001			<0.001			<0.001			<0.001			<0.001			<0.001
WT	78 (11.4%)	168 (24.5%)		56 (8.2%)	190 (27.7%)		51 (7.4%)	195 (28.4%)		91 (13.3%)	155 (22.6%)		42 (6.1%)	204 (29.7%)		37 (5.4%)	209 (30.5%)	
Mut	268 (39.1%)	172 (25.1%)		288 (42%)	152 (22.2%)		293 (42.7%)	147 (21.4%)		255 (37.2%)	185 (27%)		301 (43.9%)	139 (20.3%)		307 (44.8%)	133 (19.4%)	
1p/19q codeletion, *n* (%)						<0.001			<0.001			<0.001			<0.001			<0.001
Codel	142 (20.6%)	29 (4.2%)		125 (18.1%)	46 (6.7%)		144 (20.9%)	27 (3.9%)		141 (20.5%)	30 (4.4%)		125 (18.1%)	46 (6.7%)		135 (19.6%)	36 (5.2%)	
Non-codel	205 (29.8%)	313 (45.4%)		221 (32.1%)	297 (43.1%)		204 (29.6%)	314 (45.6%)		206 (29.9%)	312 (45.3%)		222 (32.2%)	296 (43%)		212 (30.8%)	306 (44.4%)	
OS event, *n* (%)						<0.001			<0.001			<0.001			<0.001			<0.001
Alive	252 (36.2%)	172 (24.7%)		259 (37.2%)	165 (23.7%)		270 (38.8%)	154 (22.1%)		246 (35.3%)	178 (25.6%)		261 (37.5%)	163 (23.4%)		267 (38.4%)	157 (22.6%)	
Dead	96 (13.8%)	176 (25.3%)		89 (12.8%)	183 (26.3%)		78 (11.2%)	194 (27.9%)		102 (14.7%)	170 (24.4%)		87 (12.5%)	185 (26.6%)		81 (11.6%)	191 (27.4%)	
DSS event, *n* (%)						<0.001			<0.001			<0.001			<0.001			<0.001
Alive	257 (38.1%)	174 (25.8%)		261 (38.7%)	170 (25.2%)		273 (40.4%)	158 (23.4%)		251 (37.2%)	180 (26.7%)		265 (39.3%)	166 (24.6%)		271 (40.1%)	160 (23.7%)	
Dead	83 (12.3%)	161 (23.9%)		81 (12%)	163 (24.1%)		70 (10.4%)	174 (25.8%)		90 (13.3%)	154 (22.8%)		75 (11.1%)	169 (25%)		70 (10.4%)	174 (25.8%)	
PFI event, *n* (%)						<0.001			<0.001			<0.001			<0.001			<0.001
Alive	209 (30%)	141 (20.3%)		213 (30.6%)	137 (19.7%)		220 (31.6%)	130 (18.7%)		209 (30%)	141 (20.3%)		212 (30.5%)	138 (19.8%)		219 (31.5%)	131 (18.8%)	
Dead	139 (20%)	207 (29.7%)		135 (19.4%)	211 (30.3%)		128 (18.4%)	218 (31.3%)		139 (20%)	207 (29.7%)		136 (19.5%)	210 (30.2%)		129 (18.5%)	217 (31.2%)	

### Bioinformatics Analysis

The Wilcoxon rank-sum test was used to compare MS4A expression with distinct clinicopathological features in glioma patients. Survival curves were generated by applying the Kaplan–Meier method. Cox proportional hazard regression models were used to evaluate the prognostic value of clinical factors. The correlations between MS4A expression and other genes or immune inhibitory molecules in glioma were evaluated by Spearman’s correlation and represented in a heat map form. STRING (https://string-db.org/) (Szklarczyk et al., 2019) was used to assess the interactions of six MS4A family members by conducting a PPI network analysis. Gene Ontology (GO) and Kyoto Encyclopedia of Genes and Genomes (KEGG) pathway analyses as well as gene set enrichment analysis (GSEA) (Subramanian et al., 2005) were conducted to predict biological pathways. TIMER2.0 (http://timer.cistrome.org/) (Li et al., 2020) was employed to correct the effect of tumor purity on the expression of genes and performed correlation analysis.

### Cell Culture

The human astrocytes (HA) cell line was obtained from ScienCell Research Laboratories. Human glioma cell lines such as LN229 and U251 were purchased from the Chinese Academy of Sciences Cell Bank. The cell lines were cultured in DMEM with 10% fetal bovine serum and 1% penicillin/streptomycin. The cells were maintained in a 5% CO_2_ humidified incubator at 37°C.

### Small Interfering RNA-Mediated Silencing

According to the product instructions (Ruibo company), siRNA was transfected at an siRNA concentration of 100 nM. Small interfering RNA (siRNA) target sequences for TMEM176B were as follows: si-TMEM176B#1, sense: 5′-GAG​CTT​ACA​TGC​AGA​TGC​T-3′; si-TMEM176B#2, sense: 5′-GCT​GGA​GGT​TCT​CTG​AAG​A-3′. Western blotting and RT-qPCR were used to determine the efficiency of siRNA knockdown.

### RNA Extraction and Real-Time Quantitative PCR

Total RNA was extracted from HA, LN229, and U251 cells with TRIzol Reagent (Invitrogen). The Reverse Transcription System kit (TaKaRa) was used to perform RNA reverse transcription reactions. Next, SYBR-Green (TaKaRa) and qRT-PCR analyses were used for detecting cDNA expression levels, and β-actin was used as an internal reference. Primers are shown as follows: β-actin, Forward (F): 5′-TGG​CAC​CCA​GCA​CAA​TGA​A-3′, Reverse(R): 5′-CTA​AGT​CAT​AGT​CCG​CCT​AGA​AGC​A-3′; TMEM176B, Forward (F): 5′-TGT​TGT​CCT​CTG​CGT​GAA​TAG​C-3′, Reverse (R): 5′-TTC​CTC​AGC​ATC​TGC​ATG​TAA​G-3′.

### Western Blot

Cells were lysed with protein lysis buffer containing a cocktail of protease and phosphatase inhibitors (Roche). Quantitative analysis of protein content was measured by the BCA kit (Beyotime, China) and separated using 10% sodium dodecyl sulfate-polyacrylamide gel electrophoresis. The separated proteins were transferred to nitrocellulose membranes and blocked in 5% non-fat milk. The membranes were incubated with primary antibodies overnight at 4°C. Antibodies used are given in the following: GAPDH rabbit antibody (1:1,000, cat. No. 5174; CST), TMEM176B rabbit antibody (1:200, cat. No. 19825-1-AP; Proteintech), SNAI1 rabbit antibody (1:1,000, cat. No. 180714; Abcam), vimentin rabbit antibody (1:1,000, cat. No. 5741; CST), N-cadherin rabbit antibody (1:1,000, cat. No. 13116; CST), and TNF-α rabbit antibody (1:1,000, cat. No. 6945; CST). A chemiluminescence detection system (Bio-Rad Laboratories, Inc., Hercules, CA, United States) was used to visualize the blots.

### CCK-8 Assay

Cell Counting Kit-8 (CCK-8) assay (Fdbio Science, China) was used to measure cell viability. After transfection, LN229 cells were seeded into a 96-well cell plate at a density of 2 × 10^3^ cells/well; the absorbance values were detected 0–4 days after transfection, and 10 μl of CCK-8 solution was added daily, followed by incubation for 2 h. Then, a microplate reader (Thermo) was used to measure the absorbance at 450 nm.

### Wound-Healing Assay

LN229 cells were seeded in a six-well plate and grown to 100% confluency. Using a sterile 200-μl pipette tip, a scratch was made in each cell monolayer. After washing with phosphate-buffered saline (PBS) three times, the cells were cultured with a serum-free medium and incubated in a 37°C, 5% CO_2_ incubator. Images were taken at 0 h and 48 h using the microscope (Olympus IX73). The scratch area was analyzed using ImageJ software.

### Statistical Analysis

R language (version 3.6.1), GraphPad Prism 8.0 (GraphPad Software, Inc., San Diego, CA, United States), and SPSS 17.0 (SPSS, Inc.) were used for the statistical analysis and generating figures. Two-tailed Student’s t-test and analysis of variance were performed, respectively, to compare the differences between the data of the two groups. Each experiment was repeated three times or more, and all data were presented as mean ± standard deviation (SD). Statistical significance is described as follows: ∗*p* < 0.05; ∗∗*p* < 0.01; ∗∗∗*p* < 0.001; ∗∗∗∗*p* < 0.0001.

## Results

### Transcription Levels of MS4As in Glioma Patients

**FIGURE 1:**
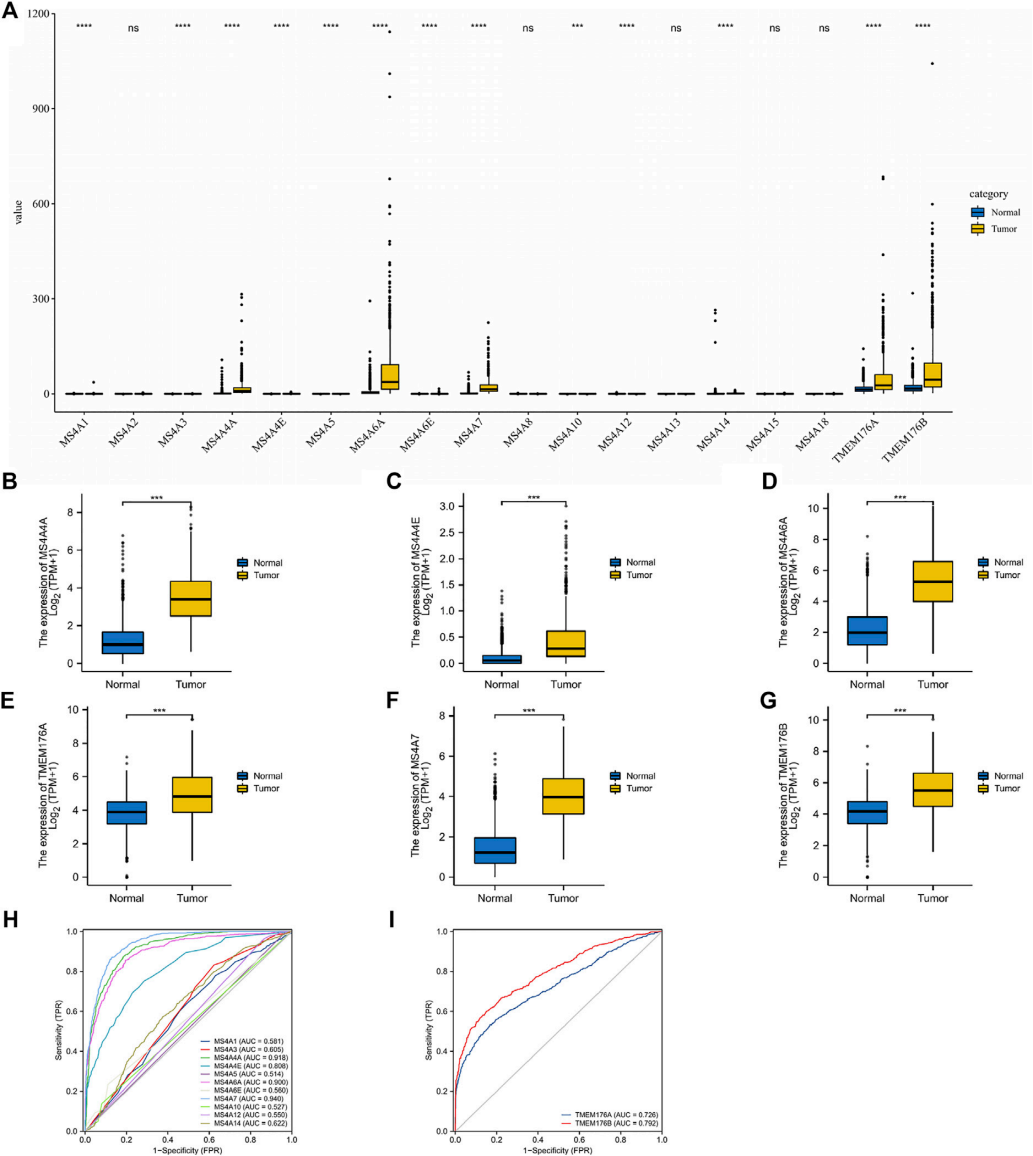
Differential expression of MS4As in glioma. **(A–G)** MS4A mRNA expression level in glioma in TCGA dataset. **(H,I)** Receiver operating characteristic (ROC) curve analysis and area under curve (AUC) statistics were used to evaluate the ability of MS4As to distinguish gliomas from normal tissues. *p*- value: 0 ≤ * < 0.05.

In total, eighteen members of the MS4A family have been identified in human cells. Based on the combined data gathered from TCGA and GTEx, we analyzed the different expression levels of MS4As between the glioma and adjacent normal tissues (Figures 1A–G). The obtained results show that the expression levels of most MS4A family members in glioma tissue were higher than those in normal tissue; however, MS4A2, MS4A8, MS4A13, MS4A15, and MS4A18 had no significant differences between the glioma and adjacent normal tissues. Subsequently, we used receiver operating characteristic (ROC) curve analysis to assess the diagnostic efficiency of the MS4A mRNA level in glioma. The result suggests that MS4A4A, MS4A4E, MS4A6A, MS4A7, TMEM176A, and TMEM176B showed good predictive power, with AUC > 0.7 (Figures 1H,I). The aforementioned results demonstrate that MS4A4A, MS4A4E, MS4A6A, MS4A7, TMEM176A, and TMEM176B might be potential biomarkers for the diagnosis of glioma. Thus, the six MS4As were included in our further study.

### MS4A Expression Is Associated With Distinct Clinicopathological Features in Glioma Patients

**FIGURE 2:**
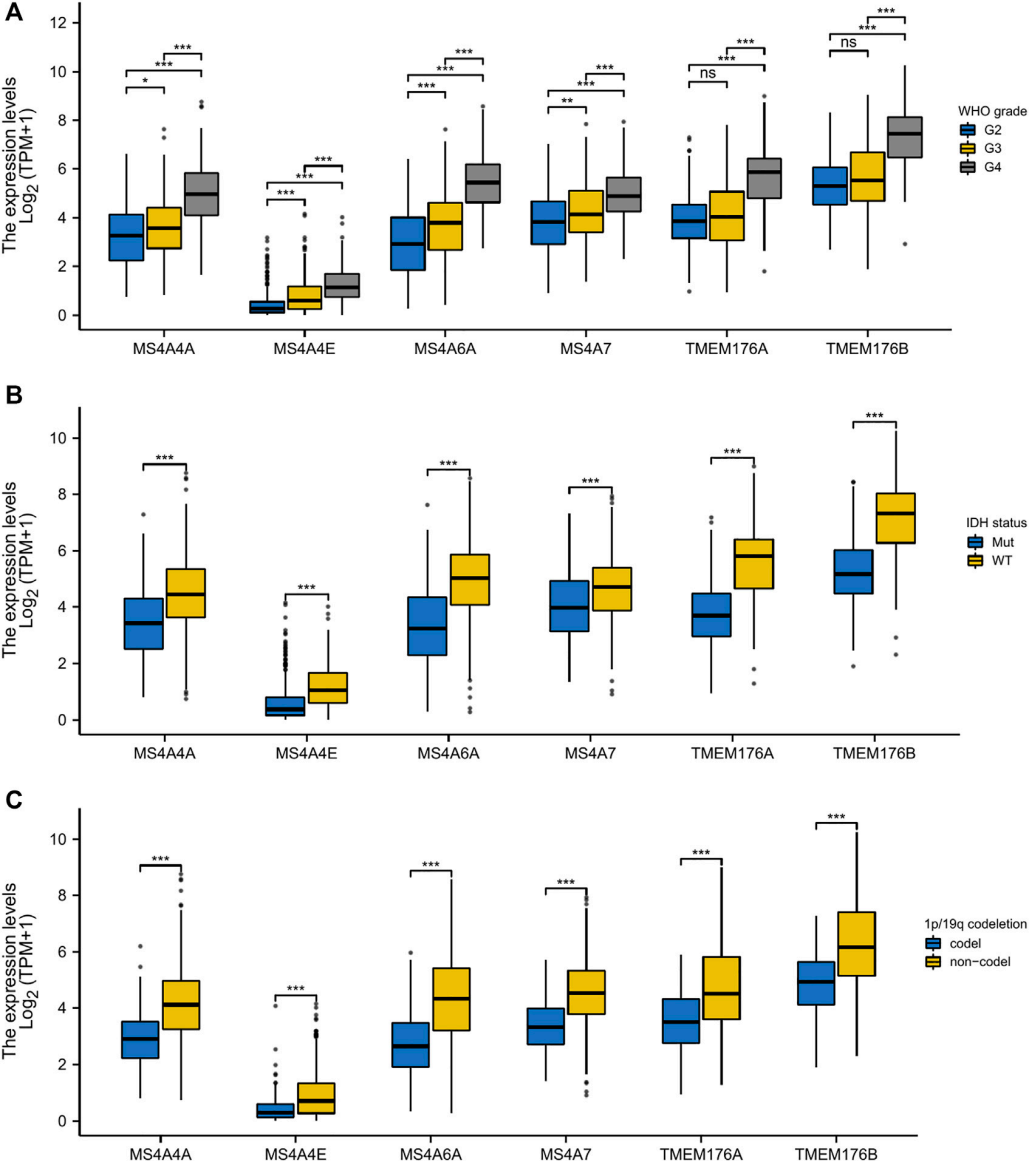
Relationship between MS4A mRNA level and clinicopathological parameters in glioma [**(A)** WHO grade; **(B)** IDH status; and **(C)** 1p/19q codeletion]. *p*- value: 0 ≤ *** < 0.001 ≤ ** < 0.01 ≤ * < 0.05.

To analyze the transcription levels of the MS4A family in the subtypes of glioma patients, TCGA databases were applied (GBM: *n* = 174; LGG: *n* = 529). According to the tumor grades, in TCGA database, compared with WHO II and III, the transcription levels of MS4A4A, MS4A4E, MS4A6A, MS4A7, TMEM176A, and TMEM176B were the highest in WHO IV (Figure 2A). IDH1/2 mutation and chromosome 1p/19q codel represent driver events during glioma tumorigenesis and are associated with better survival rates in glioma (Lv et al., 2021). Therefore, we studied the expression level of MS4As in the IDH mutant and wild type. In TCGA data, the expression level of MS4A4A, MS4A4E, MS4A6A, MS4A7, TMEM176A, and TMEM176B in IDH wild-type glioma was elevated (Figure 2B). Next, we analyzed the expression level of MS4As in 1p/19q codel and 1p/19q non-codel. In TCGA data, the expression level of MS4A4A, MS4A4E, MS4A6A, MS4A7, TMEM176A, and TMEM176B in 1p/19q non-codel glioma was elevated (Figure 2C).

### The Correlation Between MS4A Expression and the Prognosis of Gliomas

**FIGURE 3:**
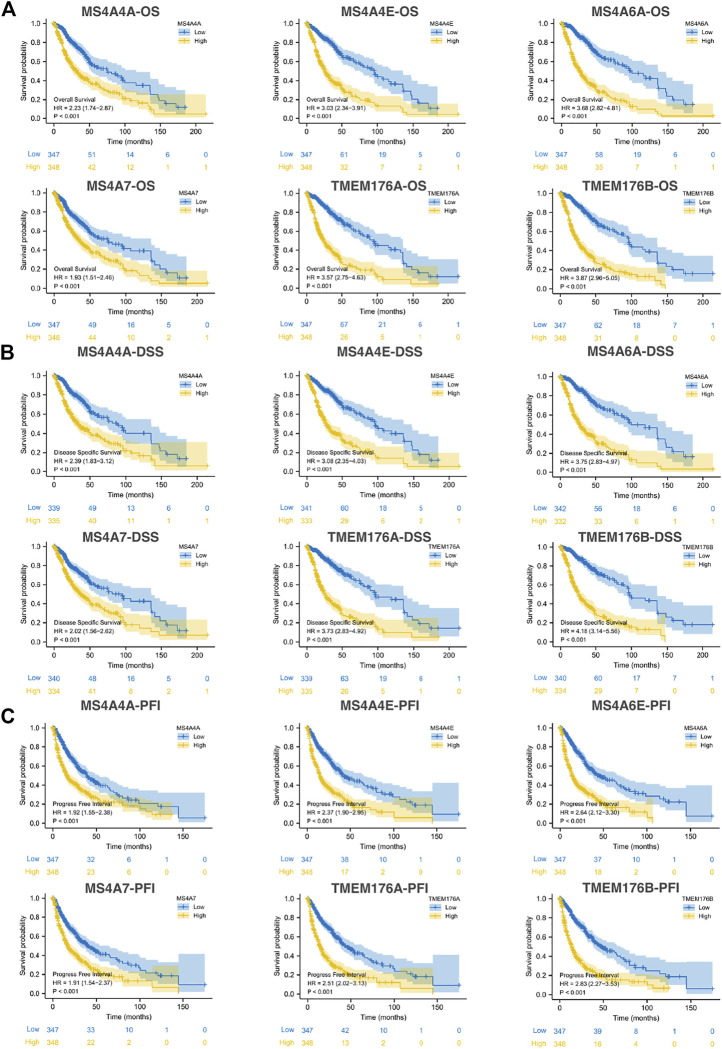
Survival curve for MS4As using data obtained from TCGA [**(A)** OS; **(B)** DSS; and **(C)** PFI].

**FIGURE 4:**
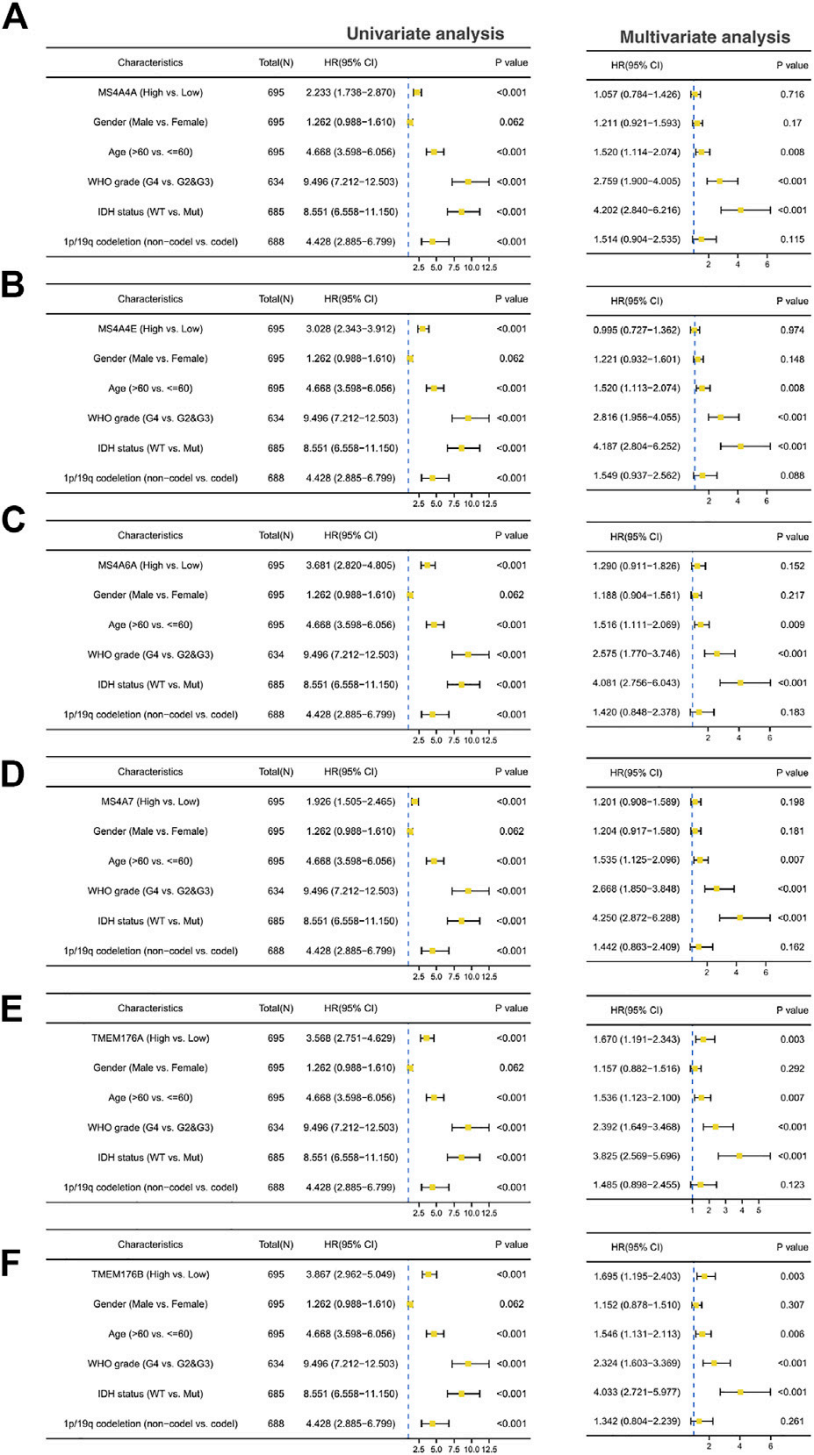
Univariate and multivariate Cox prognostic analyses of the correlation between MS4A expression with clinical–pathological factors [**(A)** MS4A4A; **(B)** MS4A4E; **(C)** MS4A6A; **(D)** MS4A7; **(E)** TMEM176A; and **(F)** TMEM176B].

**FIGURE 5:**
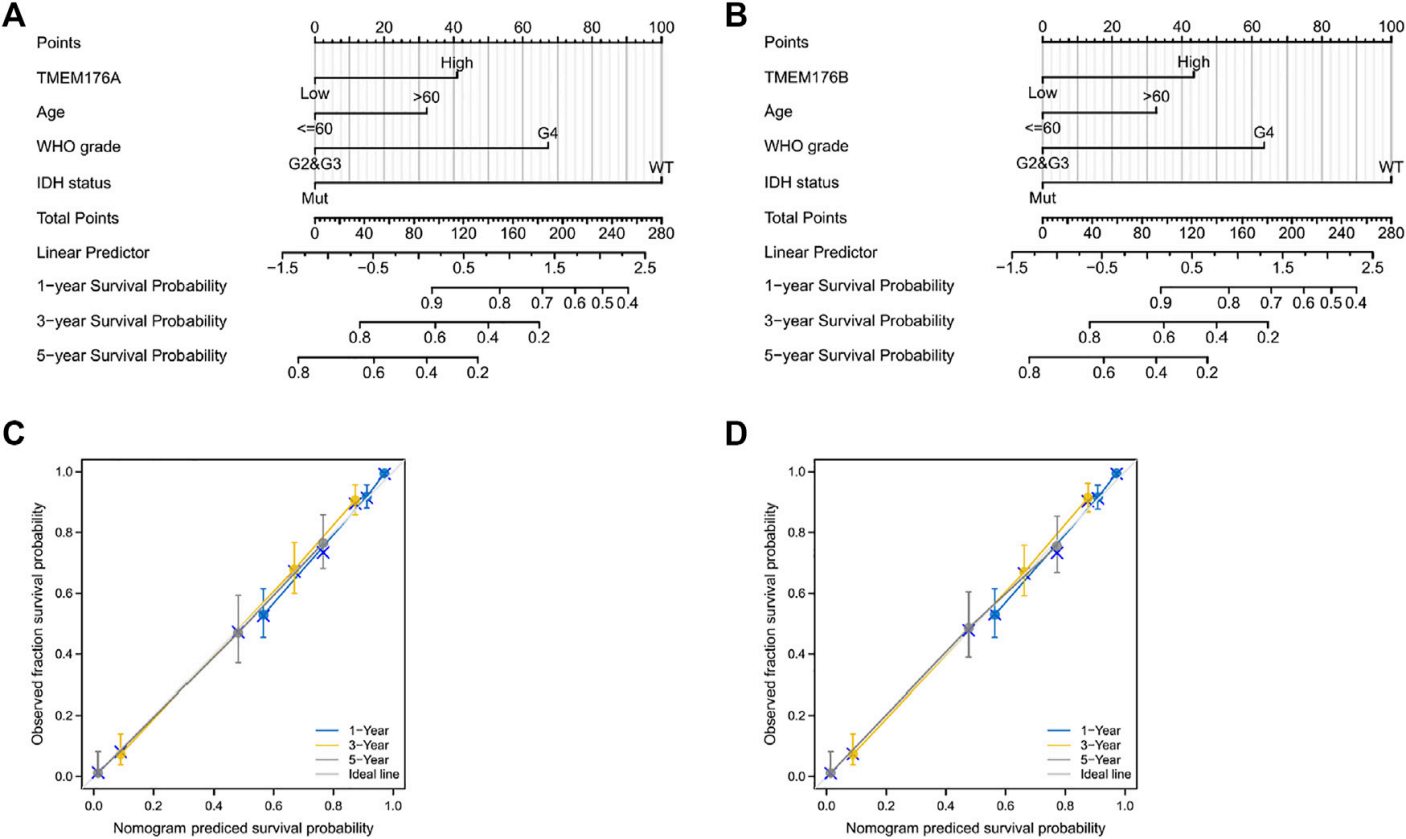
MS4A-related prognostic nomograms. **(A,B)** Nomograms for 1-, 3-, and 5-year survival; **(C,D)** calibration curve for MS4As. The abscissa is the probability of nomogram-predicted OS, and the ordinate is the observed OS.

We then performed a Kaplan–Meier survival analysis using data obtained from TCGA to investigate the prognostic value of MS4As in glioma (Figure 3). A worse prognosis was observed in the high-MS4A4A expression group than in the low-MS4A4A expression group when considering OS (overall survival), DSS (specific survival), and PFI (progression free interval). Similar results were also obtained in MS4A4E, MS4A6A, MS4A7, TMEM176A, and TMEM176B groups. Therefore, these preliminary results indicate that MS4A4A, MS4A4E, MS4A6A, MS4A7, TMEM176A, and TMEM176B are potential prognostic factors for glioma. Additionally, to evaluate the independent risk factors for prognosis of glioma, we conducted univariate and multivariate Cox analyses (Figure 4). In univariate analysis, MS4A expression in tumor cells, age, WHO grade, IDH status, and 1p/19q codeleted status was shown to be prognostic variables for the prognosis of the overall survival in glioma patients. Then, we included the prognostic variables in the univariate analysis into the multivariate analysis. We found that TMEM176A expression in tumor cells and TMEM176B expression in tumor cells, age, WHO grade, and IDH status were independent prognostic factors in glioma. Next, we established nomograms to integrate MS4As and other independent prognostic factors identified in the univariate and multivariate Cox regression analyses, including age, WHO grade, and IDH status. From these nomograms, we could obtain the total points and estimate glioma patients’ survival rates at 1-, 3-, and 5-years, making the predictive method more intuitive (Figures 5A,B). Moreover, the C-index values for the prediction model of TMEM176A and TMEM176B were 0.832 and 0.833, indicating a moderate predictive accuracy for OS in glioma. The bias-corrected curves in the calibration plots conformed well to the ideal line (the 45° line), demonstrating an excellent predictive ability (Figures 5C,D).

### Predicted Functions and Pathways of the Changes in MS4A Factors and Their Associated Genes in Glioma Patients

**FIGURE 6:**
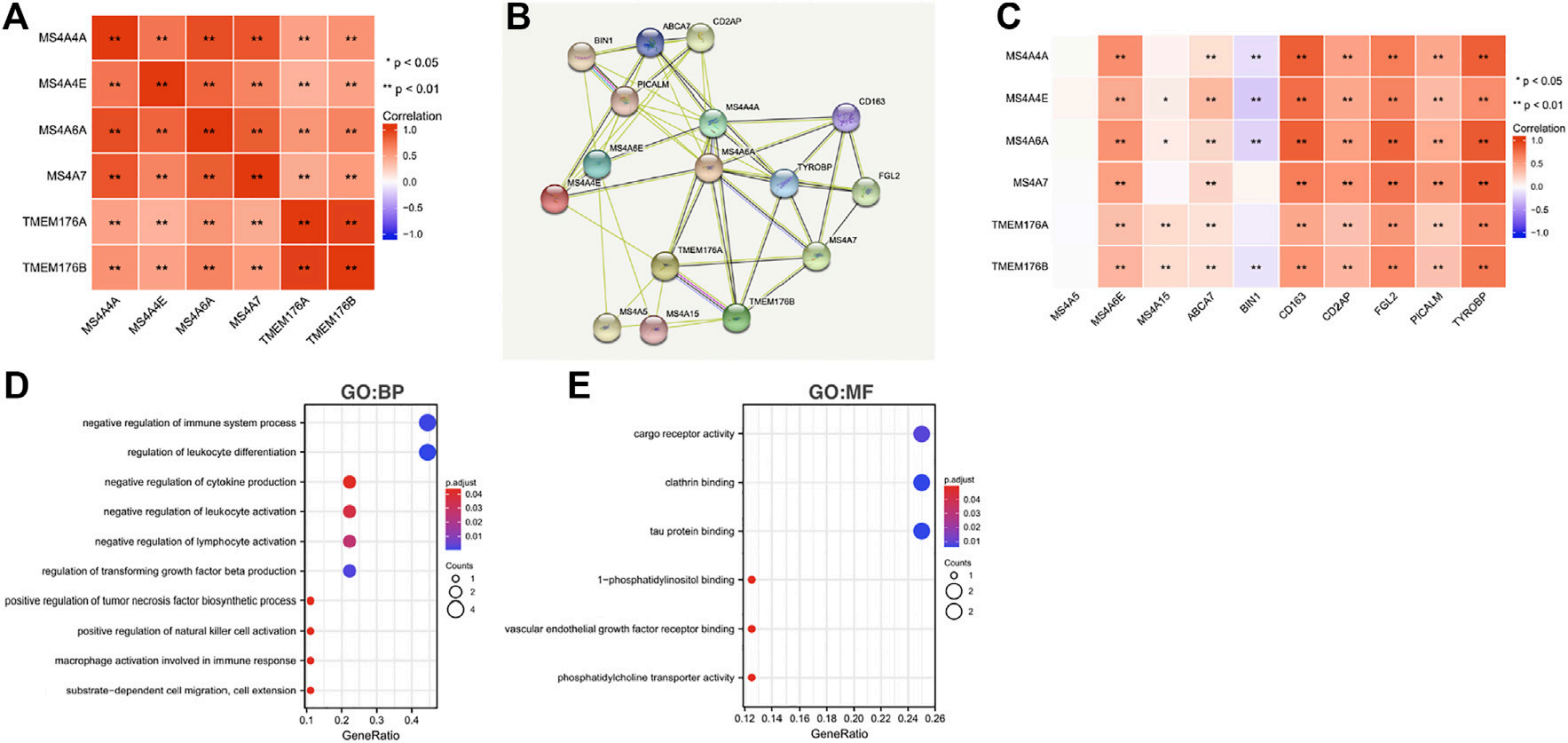
Predicted pathways and correlations of the MS4A expression in glioma. **(A)** Spearman’s correlation coefficients were used to study the correlations among MS4As; **(B)** PPI network for MS4As was constructed in STRING; **(C)** GO terms related to biological processes (BPs) are shown in a bubble chart; **(D)** GO terms related to molecular functions (MFs) are shown in a bubble chart; **(E)** Spearman’s correlation coefficients were used to study the association between MS4As and ten key genes.

**FIGURE 7:**
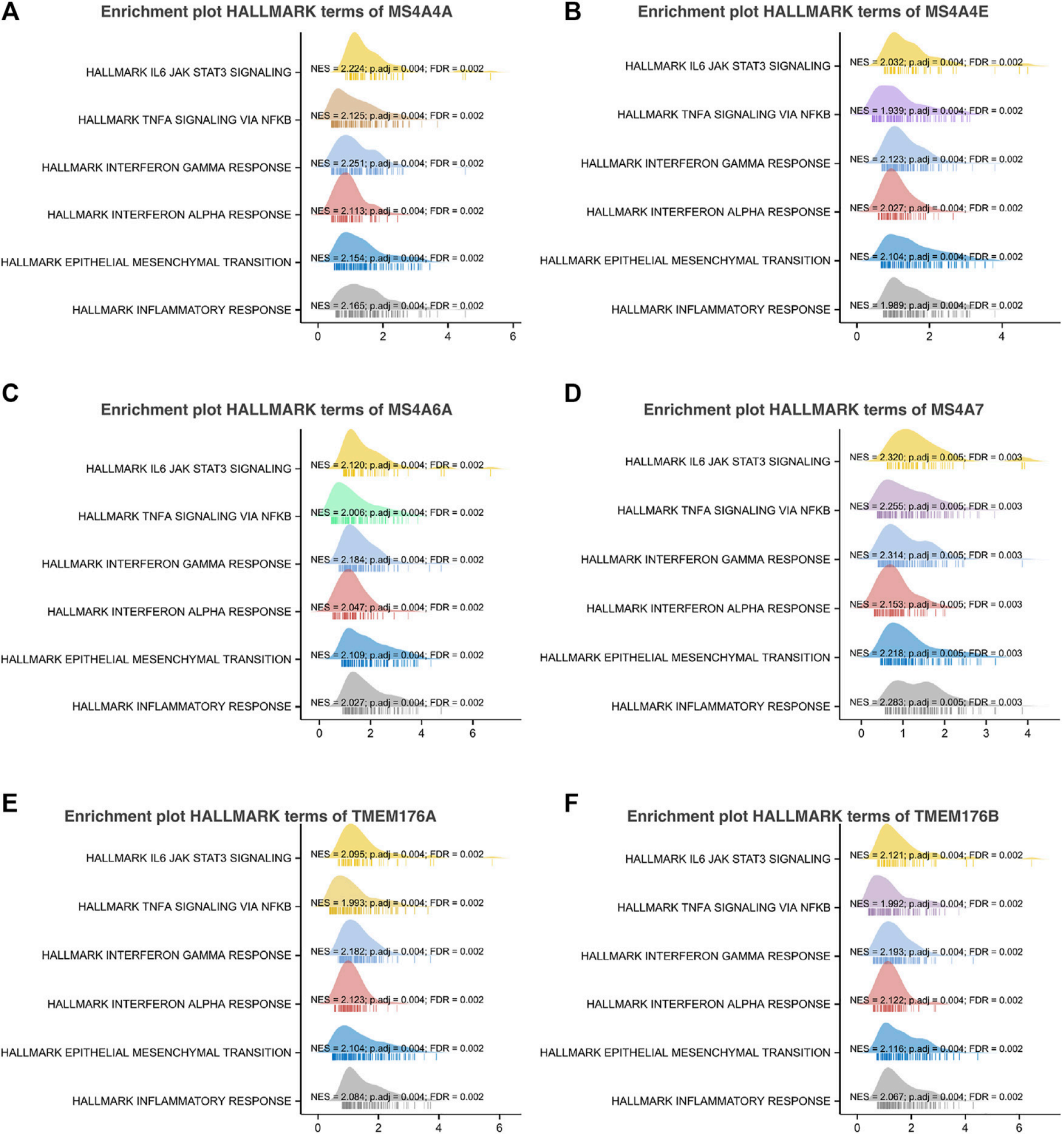
Gene set enrichment analysis of associated genes with MS4As [**(A)** MS4A4A; **(B)** MS4A4E; **(C)** MS4A6A; **(D)** MS4A7; **(E)** TMEM176A; and **(F)** TMEM176B].

To further understand the MS4A family, first, we used Spearman’s correlation coefficients to study the correlations among the expression levels of the MS4A family in glioma using data obtained from TCGA (Figure 6A). The results indicated that there existed positive correlations among MS4As (*p* < 0.05). Next, we performed PPI network analysis of the six MS4A family members *via* STRING to explore potential protein interactions and to seek network-related genes, and the results are shown in Figure 6B. The functions of MS4As and their associated genes were predicted by analyzing Gene Ontology (GO) and Kyoto Encyclopedia of Genes and Genomes (KEGG) in the Database for Annotation, Visualization and Integrated Discovery (DAVID) (https://david.ncifcrf.gov/summary.jsp). Biological process (BP) enrichment terms showed that MS4As and their associated genes were significantly associated with regulation of leukocyte differentiation, negative regulation of immune system process, regulation of transforming growth factor beta production, negative regulation of lymphocyte activation, negative regulation of leukocyte activation, negative regulation of cytokine production, substrate-dependent cell migration, cell extension, macrophage activation involved in immune response, positive regulation of natural killer cell activation, and positive regulation of tumor necrosis factor biosynthetic process (Figure 6D). MF enrichment showed that MS4As and their associated genes were significantly associated with tau protein binding, clathrin binding, cargo receptor activity, phosphatidylcholine transporter activity, vascular endothelial growth factor receptor binding, and 1-phosphatidylinositol binding (Figure 6E). Then, we obtained ten key genes in the PPI network (Figure 6B), which participated in the aforementioned immune mechanisms, and their information is shown in heatmaps (Supplementary Figures S1A–F). By using Spearman’s correlation coefficients to analyze associations between these ten genes and *MS4As*, we studied that seven genes were positively correlated with *MS4As:MS4A6E*, *ABCA7*, *CD163*, *CD2AP*, *FGL2*, *PICALM*, and *TYROBP* (Figure 6C). Clearly, the result of GO analysis revealed that MS4As were related to the immune response. We believe that MS4As may play an immunomodulation role in glioma. Many signaling pathways contribute to tumor initiation and progression, and the poor prognosis of high-MS4A expression may be related to the numerous signaling pathways activated in glioma. Thus, we used GSEA to recognize signaling pathways involved in glioma between low- and high-MS4A expression cohorts. Several HALLMARKER items were enriched in the group of high-MS4A expression (*p* < 0.05, FDR < 0.05), including TNF α *via* NF-kB signaling, IL6/JAK/STAT3 signaling, IFN- γ response, IFN- α response, epithelial–mesenchymal transition (EMT), and inflammatory response (Figure 7). The result indicated that they might play an important role in the development of glioma.

### MS4A Correlation With Immune Infiltration and Immune Inhibitory Molecule Level

**FIGURE 8:**
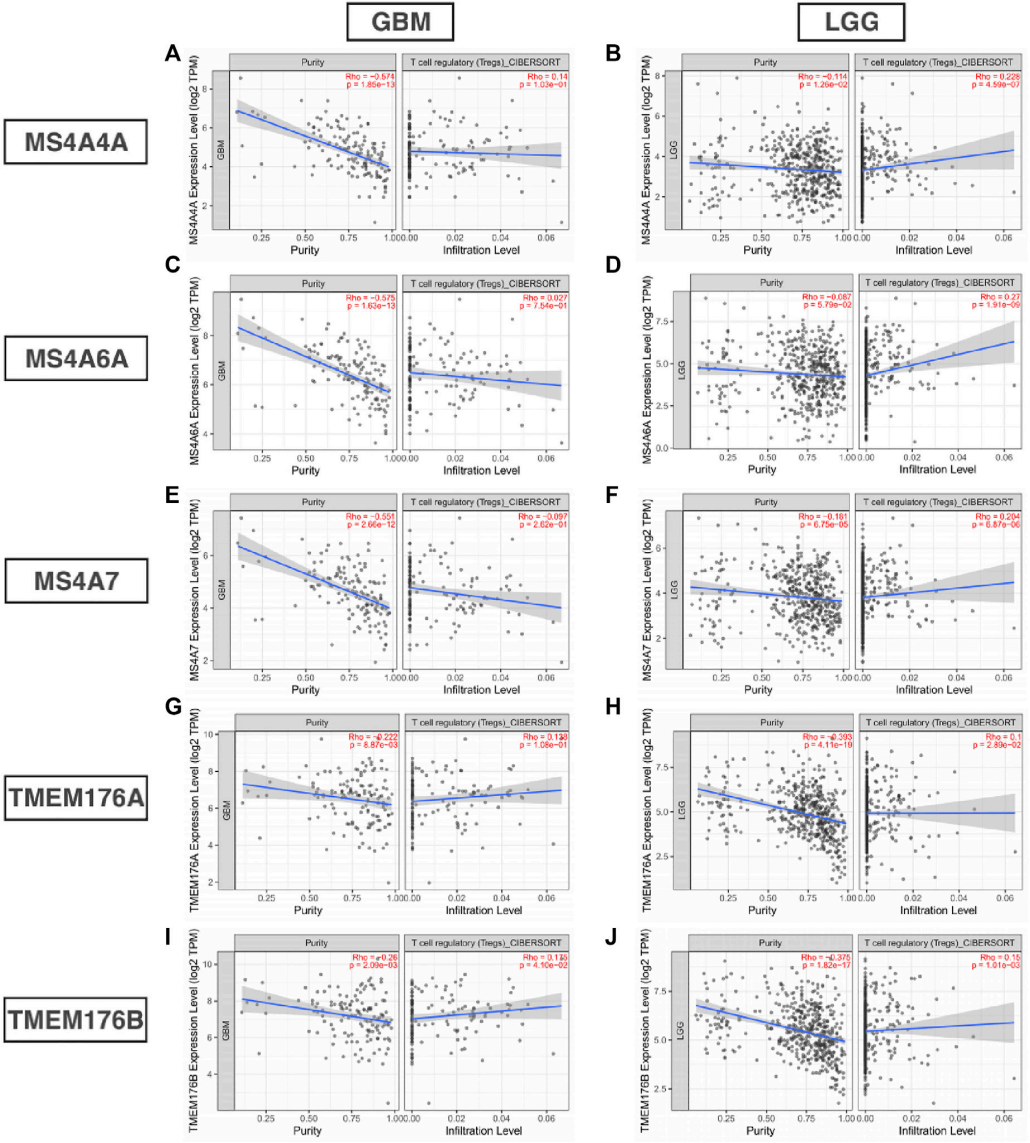
Correlation between MS4As and the level of Treg infiltration in GBM and LGG **(A–J)**.

**FIGURE 9:**
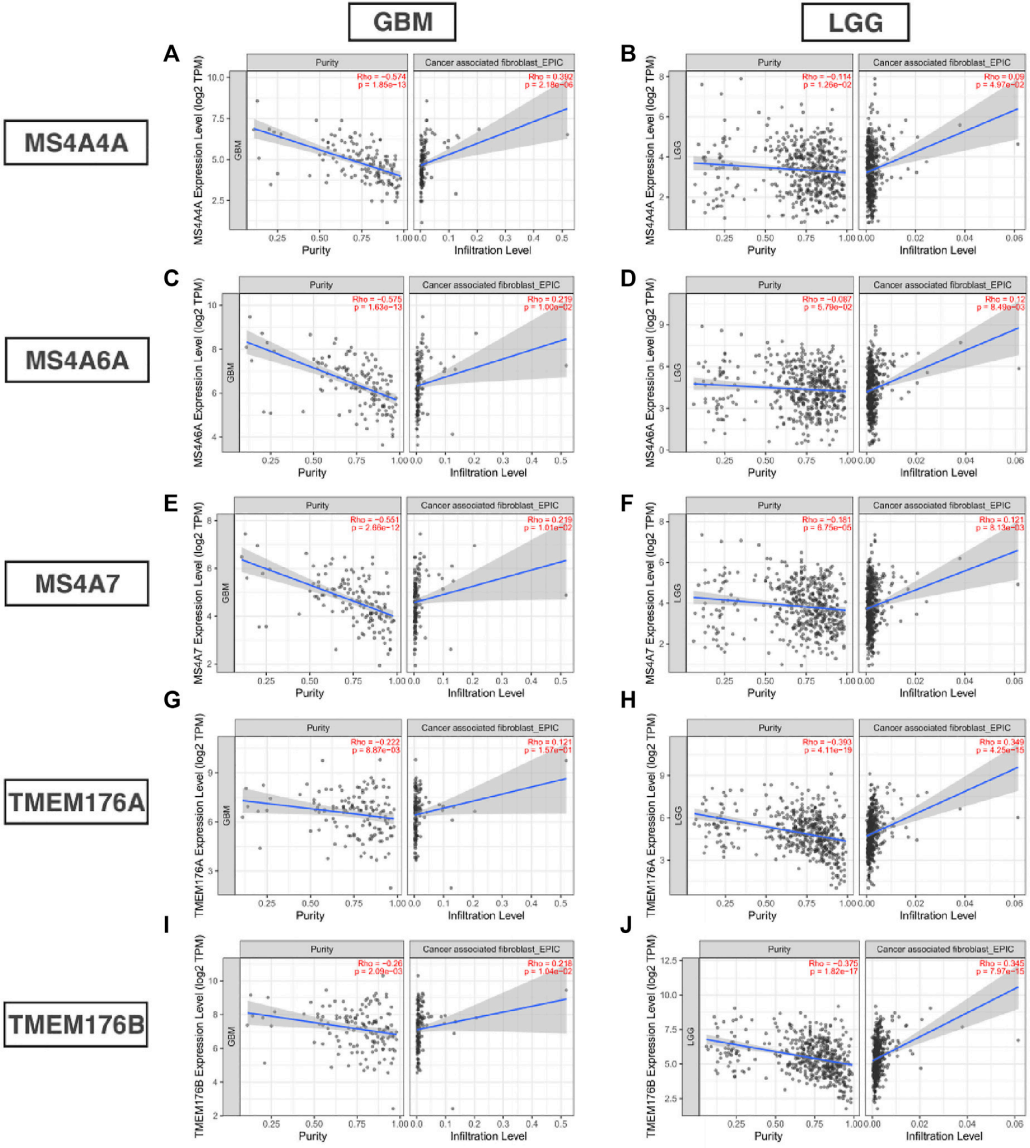
Correlation between MS4As and the level of CAF infiltration in GBM and LGG **(A–J)**.

Infiltrating immune cells are important components of the tumor microenvironment and are frequently associated with tumor behavior and patient outcomes. Since GO analysis and KEGG analysis revealed that MS4As were related to the immune response, we used TIMER to analyze the correlation between MS4As and infiltrating immune cell gene markers in GBM and LGG (Supplementary Table S1). After reviewing the previous studies, we selected gene markers of immune cells, including B cells, T cells (general), CD8^+^ T cells, Tregs (regulatory T cells), T-cell exhaustion, neutrophils, monocytes, M1 and M2 macrophages, TAMs (tumor-associated macrophages), and CAFs (cancer-associated fibroblasts). We found that MS4As were significantly associated with marker sets of Tregs, T-cell exhaustion, M2 macrophages, TAMs, and CAFs after purity adjustment (*p* < 0.05). Since Tregs and CAFs play an important role in promoting tumor progression, we also analyzed the correlation among MS4As, Tregs, and CAFs. The result showed that high-MS4A expressions were associated with high levels of Treg infiltration in LGG (*p* < 0.05); however, MS4A expressions were not significantly related to the levels of Treg infiltration in GBM (Figure 8). Meanwhile, high-MS4A expressions were associated with high levels of CAF infiltration in GBM and LGG (*p* < 0.05) (Figure 9). Tregs and CAFs play key roles in the negative regulation of the immune response, leading to tumor progression due to immune evasion. Thus, the high expression of MS4As might contribute to immunotherapy resistance.

**FIGURE 10:**
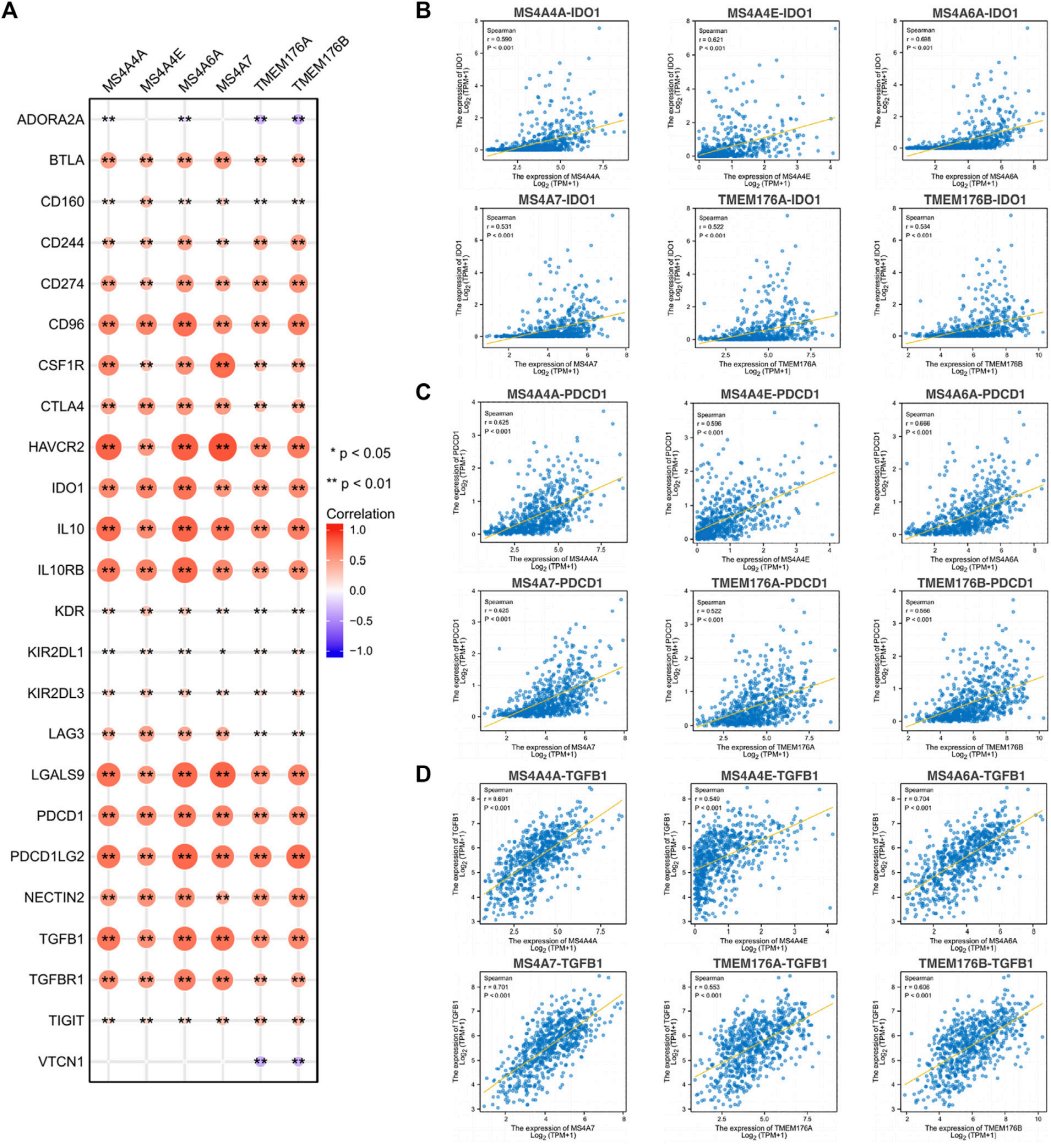
Correlations between MS4As and immune inhibitory molecules. **(A)** MS4A expression is highly correlated with immune inhibitory molecules in glioma. The color and size of the circles represent the R-value of Spearman’s correlation. **(B–D)** Scatter plots showing the strong association of MS4A levels with *IDO1*, *PDCD1*, and *TGFB1*. Correlation coefficients were classified as weak (0.1 ≤ *r* < 0.3), moderate (0.3 ≤ *r* < 0.5), or strong (*r* ≥ 0.5).

To further explore the potential immune mechanism of MS4As in glioma, we analyzed correlations between MS4As with twenty-four immune inhibitory molecules, including ADORA2A, BTLA, CD160, CD244, CD274 (PDCD1LG1), CD96, CSF1R, CTLA4, HAVCR2, IDO1, IL10, IL10RB, KDR, KIR2DL1, KIR2DL3, LAG3, LGALS9, PDCD1, PDCD1LG2, NECTIN2, TGFB1, TGFBR1, TIGIT, and VTCN1 (Figure 10). We found that MS4As were strongly positively correlated with a majority of immune inhibitory molecules, such as CD96, HAVCR2, IDO1, IL10, IL10RB, LGALS9, PDCD1, PDCD1LG2, and TGFB1 (*r* > 0.5, *p* < 0.001). Among them, IDO1, PDCD1, and TGFB1 are promising immune-modulatory targets that are in the focus of current clinical research in glioblastoma (Shadbad et al., 2021). These results indicated that MS4As were closely related to common immunotherapeutic targets for glioma and that MS4As may be new immunotherapeutic targets.

### Knockdown of TMEM176B Suppresses Malignant Properties of Glioma Cells

**FIGURE 11:**
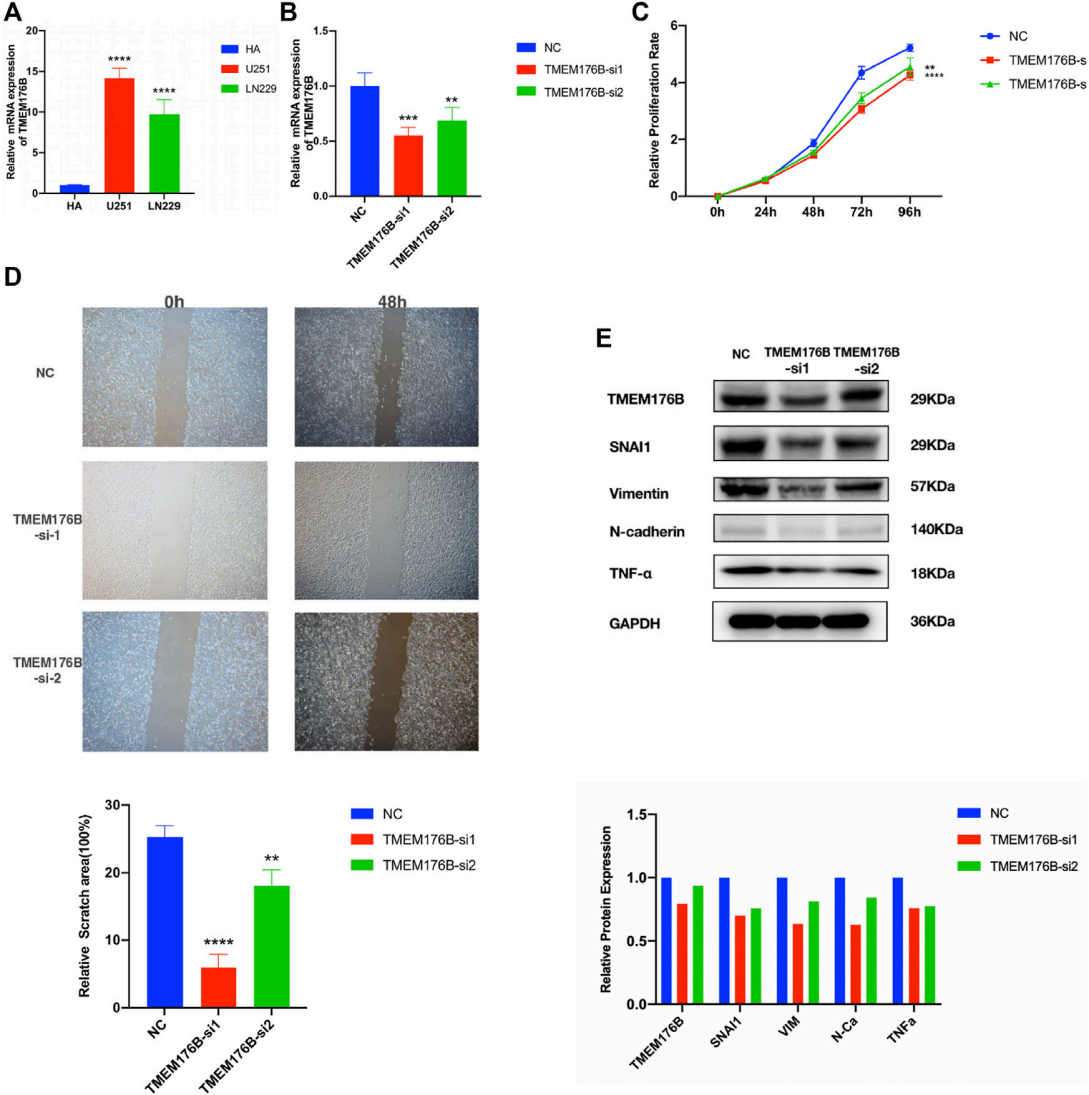
Biological function investigation and *in vitro* verification of TMEM176B in glioma. **(A)** Relative mRNA expression levels of TMEM176B in the human astrocytes (HA) cell line and two glioma cell lines were detected by RT-qPCR. **(B)** Efficiency of TMEM176B siRNAs was detected by RT-qPCR. **(C)** CCK-8 assay was performed to test the proliferation of LN229 cells. **(D)** Wound-healing assay was adopted to determine the migration of LN229 cells. Lower panel, bar graphs representing quantification of the scratch area. **(E)** Western blot analysis was adopted to detect the expression of related proteins of tumor progression. Lower panel, bar graphs representing quantification of Western blot bands. *p < 0.05; **p < 0.01; ***p < 0.001; ****p < 0.0001.

In order to verify the results of aforementioned bioinformatics analysis, we further analyzed the TMEM176B mRNA expression levels in glioma and normal brain cell lines by real-time qPCR. TMEM176B was highly expressed in various glioma cell lines (U251 and LN229). The expression levels were significantly higher than those in the normal brain cell line (HA) (Figure 11A). To examine the effect of TMEM176B in glioma cell lines, LN229 was successfully transfected with si-TMEM176B to knockdown the expression of TMEM176B and verified by real-time qPCR (Figure 11B) and Western-blot analysis (Figure 11E). First of all, the CCK-8 assay was used to measure the proliferation of siRNA-transfected cells. The LN229 cell lines, treated with si-TMEM176B #1 and #2, revealed the lower proliferative ability than the negative control groups (Figure 11C). In addition, we found that TMEM176B knockdown caused an apparent suppression of cell migration in LN229 lines by cell scratch assays (Figure 11D). Subsequently, we detected the protein levels of TNF-α and several EMT-related markers, such as SNAI1, vimentin, and N-cadherin. The results indicated that TMEM176B knockdown led to decreased protein levels of TNF-α, SNAI1, vimentin, and N-cadherin in the LN229 cell line. In conclusion, these results demonstrated that the knockdown of TMEM176B protein inhibited the proliferation and migration of glioma cells.

## Discussion

Glioma is an invasive and highly diffuse brain tumor (Greenall et al., 2015). At present, the prognosis of glioma patients is very poor, even with the use of multimodal treatment strategies. In fact, the median survival period after diagnosis of glioma patients is still approximately 15 months, and long-term survival is unsatisfactory (Wen et al., 2020). Therefore, it is urgent to explore new prognostic biomarkers and personalized treatment strategies for this disease. In this study, six members of the MS4A family including MS4A4A, MS4A4E, MS4A6A, MS4A7, TMEM176A, and TMEM176B were identified as potential diagnostic or prognostic biomarkers. In addition, most of them were correlated with tumor immune infiltration markers in glioma.

The membrane-spanning 4A (MS4A) family includes 18 members with a tetraspan structure in humans and is able to regulate cell activation by acting as ion channels or by modulating the signaling of other immune receptors (Howie et al., 2009; Mattiola et al., 2021). Previous studies revealed that the MS4A family members play an important role in different pathological settings. As cell membrane proteins, MS4A family members are found to participate in the regulation of calcium signaling, which have been widely discussed in neurodegeneration and AD (Ma et al., 2015). MS4A4A was considered a marker of macrophages and has been associated with autoimmune conditions, such as rheumatoid arthritis (Mattiola et al., 2019), cutaneous systemic sclerosis (Rice et al., 2015), polyangiitis (Ishizu et al., 2013), and Kawasaki disease (Guo et al., 2020). The expression of the MS4A family members in several cancers has been studied, suggesting that some members are related to the poor prognosis in cancers (Cuajungco et al., 2012; He et al., 2017; Sun et al., 2018). However, studies on their potential functional mechanism in glioma were elusive.

In our research, we used public cancer databases for data mining and found that most of the MS4A family genes exhibited high expression in glioma tissue compared with those in normal tissue; however, MS4A4A, MS4A4E, MS4A6A, MS4A7, TMEM176A, and TMEM176B showed good predictive power using ROC curve analysis. Thus, the six MS4As were included in our further study. Next, we learnt that an elevated MS4A expression in glioma is associated with various clinicopathological parameters (age, WHO grade, IDH status, and 1p/19q codeletion) and overall survival. Age is an independent prognostic indicator and is positively correlated with a poor prognosis in glioma (Sasaki et al., 2018). IDH mutations are considered glioma biomarkers and are generally associated with a better prognosis among glioma patients (Waitkus et al., 2016). Gliomas with 1p/19q codeletion have a favorable prognosis, and it is also a marker of chemotherapeutic response (Bush and Butowski, 2017). A high WHO grade (III or IV) may be associated with poor outcomes (Weller et al., 2015). The Cox proportional hazards regression model indicates TMEM176A and TMEM176B expressions in tumor cells are independent prognostic indicators of glioma. Meanwhile, we developed novel nomograms using factors identified in the univariate and multivariate Cox regression analyses. The C-index values and calibration plots suggested that the nomogram effectively predicts 1-, 3-, or 5-year survival for patients with glioma. In brief, these findings suggest that TMEM176A and TMEM176B are prognosis-related biomarkers in glioma.

To further explore the possible mechanism of MS4As in the progression of glioma, we performed PPI analysis, GO and KEGG pathway analysis, and GSEA analysis. GO and KEGG pathway analysis revealed that MS4As were related to immune response. In addition, GSEA concluded that MS4As were involved in several tumor-related pathways, including TNF-α *via* NF-kB signaling, IL6/JAK/STAT3 signaling, IFN- γ response, IFN-α response, and epithelial–mesenchymal transition (EMT). It has been recently reported that inflammation plays a crucial function in the occurrence, development, and prognosis of glioma (Reynés et al., 2011; Yeung et al., 2013; Michelson et al., 2016). TNF-α is one of the major regulators of inflammation and is strongly correlated with progression and clinical aggressiveness in glioma (Hwang et al., 2016). IL-6/JAK/STAT3 signaling is able to drive the proliferation, survival, invasiveness, and metastasis of tumor cells, leading to a poor clinical prognosis in many types of cancers (Johnson et al., 2018). EMT is determined to be strongly related to glioma malignancies (Iser et al., 2017). We speculate that MS4As may play a tumor-promoting role through these pathways, which can interpret why the high expression of MS4As is closely associated with the poor prognosis in glioma. The experiments were performed in *in vitro* and partly verified our findings. Knockdown of TMEM176B suppresses malignant properties of glioma cells.

In recent years, it has been recognized that immune cells may play a key role in suppressing the tumor or providing support for tumor growth (Domingues et al., 2016). However, no studies have assessed the relationship between MS4As and immune infiltration in glioma. Using the TIMER platform, we explored the association between MS4As and gene markers of immune cells in GBM and LGG. The results suggest that MS4A expression has strong correlations with marker sets of Tregs, T-cell exhaustion, M2 macrophages, TAMs, and CAFs. In tumor immune microenvironments, regulatory T cells (Tregs) are associated with tumor progression and reduced survival in cancer patients, by hindering immune responses and promoting immune evasion (Knochelmann et al., 2018). The exhaustion of T cells is a major cause of inefficient antitumor immunity (Wherry and Kurachi, 2015). We noticed that a high expression level of MS4As is positively correlated with multiple key genes of exhausted T cells including *PD-1*, *CTLA-4*, and so on, which are current targets for immunotherapy. Tumor-associated macrophages (TAMs) are an important cell population in cancers and promote tumor growth, metastasis, and neovascularization (Zhu et al., 2017). Similarly, the M2 phenotype of microglia promotes tumor cell immune evasion, invasion, proliferation, and angiogenesis (Grabowski et al., 2021). Some review articles summarized that cancer-associated fibroblasts (CAFs) not only promote tumor cell proliferation, migration, and invasion but also affect antitumor immunity (Piersma et al., 2020). Moreover, further investigations for correlations between MS4As and immune infiltration level showed that high MS4A expressions were associated with high levels of Tregs infiltration in LGG as well as high MS4A expressions were associated with high levels of CAF infiltration in GBM and LGG. This provided further evidence that MS4As play major roles in the regulation of tumor microenvironment, which was a possible mechanism for MS4As leading to the poor prognosis of glioma patients.

After assessing the correlation between MS4A expression levels and immunoinhibitors, we found a strong correlation between MS4As and *IDO1*, *PDCD1*, or *TGFB1*. Indoleamine 2,3-dioxygenase 1 (*IDO1*) is known to cause immunosuppression through breakdown of tryptophan in the tumor microenvironment. A study showed the increased therapeutic efficacy of two-fraction radiotherapy in conjunction with *IDO1* inhibition in a syngeneic rat glioblastoma model (Ahlstedt et al., 2020). Meanwhile, two highly selective *IDO1* inhibitors, PF-06840003 and BGB-5777, have demonstrated promising antitumor activity in pre-clinical models (Gomes et al., 2018; Ladomersky et al., 2018). Programmed cell death 1 (*PDCD1*) is an inhibitory receptor expressing mainly on activated T cells (Patsoukis et al., 2020). An engagement of *PDCD1* with its ligands eventually leads to apoptosis of activated T cells. Immune checkpoint monotherapy targeting the PD-1/PD-L1 axis has limited success in recurrent GBM (Checkmate-143 trial; NCT02017717) (Filley et al., 2017). Moreover, the therapeutic effect of anti-PD-1 therapy for newly diagnosed GBM is currently being investigated in two randomized phase III clinical trials (NCT02617589 and NCT02667587). Transforming growth factor-β (TGF-β) is a multitasking cytokine which induces immune tolerance by regulating multiple types of immune cells (Mirshafiey and Mohsenzadegan, 2009; Ahmadi et al., 2019). There have been reports that reducing TGF-β signaling by inhibiting mRNA translation through antisense oligonucleotides delays the growth of experimental gliomas (Papachristodoulou et al., 2019). Encouragingly, a clinical trial (NCT00431561) showed that targeting the TGF-β pathway by using antisense oligonucleotides improves disease prognosis when combined with chemotherapy (Han et al., 2015). In this study, we identified MS4As as potential immunotherapeutic targets. As seen in Figure 10, MS4As had a high concordance with prominent immune checkpoint molecules, including CD96, HAVCR2, IDO1, IL10, IL10RB, LGALS9, PDCD1, PDCD1LG2, TGFB1, and TGFBR1, suggesting their synergistic roles in regulating the immune response within the tumor microenvironment. These findings open up new possibilities for combination therapy in glioma.

## Conclusion

In summary, our study indicates that in general the increased expression of MS4A4A, MS4A4E, MS4A6A, MS4A7, TMEM176A, or TMEM176B is a poor prognostic factor. By using the GO and KEGG pathway and GSEA, we studied the mechanisms that may mediate the role of MS4As in glioma development. We also found that MS4As were involved in the inflammatory and immune responses and were correlated with immune checkpoint molecules. These findings shed more light on the complexity and heterogeneity of glioma biological properties, and further mechanistic studies are needed to validate our findings.

## Data Availability

The original contributions presented in the study are included in the article/Supplementary Material; further inquiries can be directed to the corresponding authors.
