# Supplementary figures and images for: Comprehensive Analysis of Expression and Prognostic Value of MS4As in Glioma
Source: Front Genet. 2022 Jun 6;13:795844. doi: 10.3389/fgene.2022.795844 (PMC9207330; doi:10.3389/fgene.2022.795844)

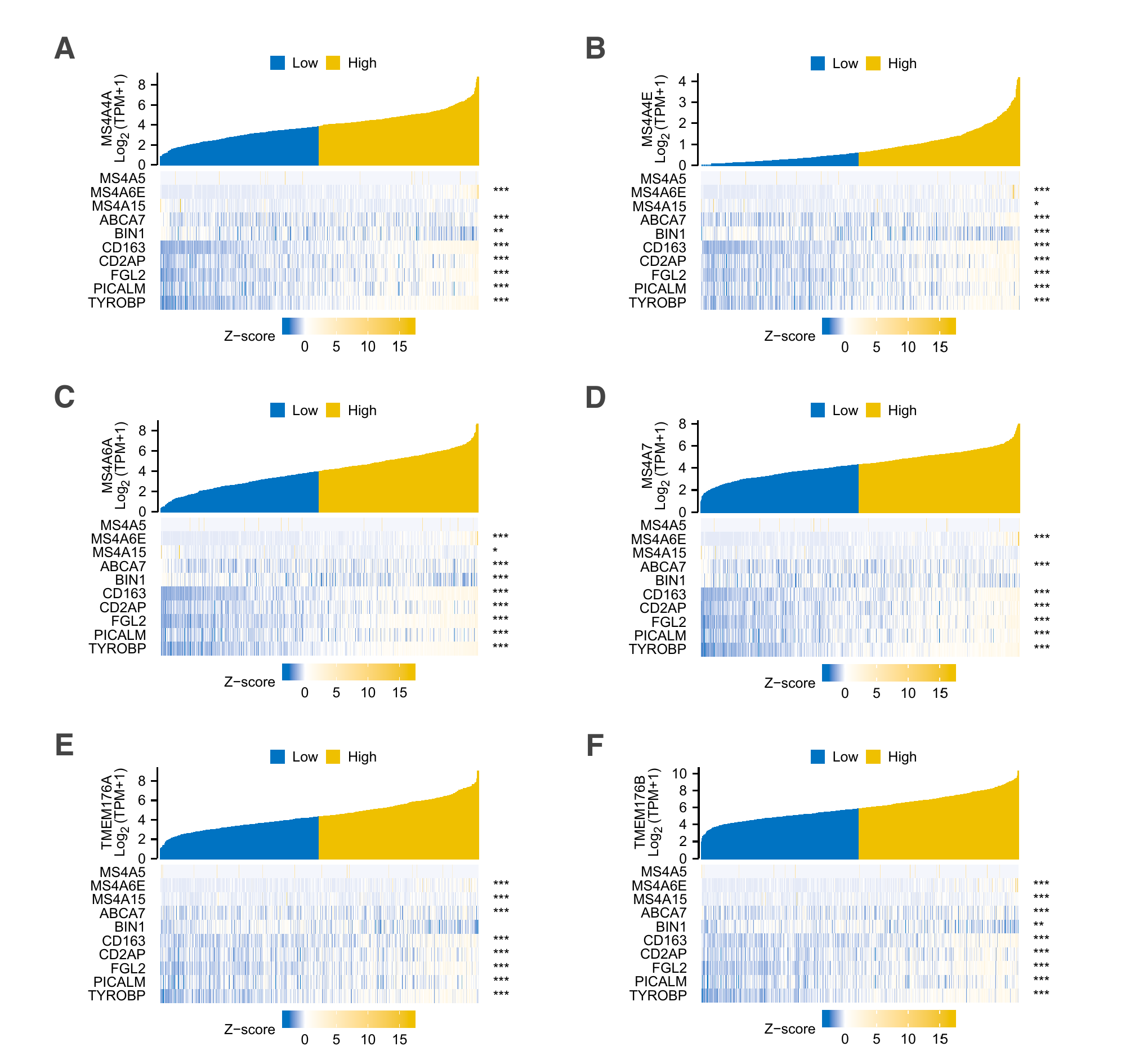

Supplement: Supplementary file 1 [file Image1.TIFF]
