# Supplementary material for: Comprehensive Analysis of Expression and Prognostic Value of MS4As in Glioma
Source: Front Genet. 2022 Jun 6;13:795844. doi: 10.3389/fgene.2022.795844 (PMC9207330; doi:10.3389/fgene.2022.795844)
Supplement: Supplementary file 2 [file Table1.DOCX]

Supplementary Table 1

Correlation analysis between MS4As and markers of immune cells in GBM and LGG. Cor, R-value of Spearman’s correlation.

| Immune cell | Gene markers | MS4A4A-GBM | | | | MS4A4A-LGG | | | | |
| --- | --- | --- | --- | --- | --- | --- | --- | --- | --- | --- |
|  |  | none | | purity | | none | | purity | | |
|  |  | cor | p | cor | p | cor | p | cor | p | |
| B cell | CD19 | 0.207 | 1.03e-02 | 0.194 | 2.33e-02 | 0.332 | 8.9e-15 | 0.295 | 3.89e-05 | |
|  | CD79A | 0.308 | 1.09e-04 | 0.343 | 4.17e-05 | 0.409 | 2.87e-22 | 0.415 | 2.83e-21 | |
| T cell (general) | CD3D | 0.585 | 2.12e-15 | 0.441 | 7.11e-08 | 0.42 | 1.66e-23 | 0.414 | 3.52e-21 | |
|  | CD3E | 0.555 | 0e+00 | 0.455 | 2.30e-08 | 0.464 | 6.38e-29 | 0.473 | 4.99e-28 | |
|  | CD2 | 0.603 | 0e+00 | 0.489 | 1.34e-09 | 0.472 | 5.3e-30 | 0.476 | 1.84e-28 | |
| CD8+ T cell | CD8A | 0.315 | 8.04e-05 | 0.238 | 5.08e-03 | 0.069 | 1.2e-01 | 0.051 | 2.66e-01 | |
|  | CD8B | 0.419 | 9.64e-08 | 0.345 | 3.72e-05 | 0.231 | 1.1e-07 | 0.214 | 2.34e-06 | |
| Treg | FOXP3 | 0.224 | 5.56e-03 | 0.149 | 8.16e-02 | -0.175 | 6.61e-05 | -0.157 | 5.38e-04 | |
|  | CCR8 | 0.489 | 1.42e-10 | 0.457 | 2.02e-08 | 0.204 | 2.98e-06 | 0.209 | 4.24e-06 | |
|  | STAT5B | -0.299 | 1.82e-04 | -0.124 | 1.49e-01 | 0.134 | 2.24e-03 | 0.148 | 1.17e-03 | |
|  | TGFB1 | 0.418 | 1.03e-07 | 0.316 | 1.71e-04 | 0.624 | 0e+00 | 0.62 | 4.10e-52 | |
| T cell exhaustion | PD-1 | 0.449 | 8.54e-09 | 0.42 | 3.23e-07 | 0.514 | 3.64e-36 | 0.515 | 9.15e-34 | |
|  | CTLA4 | 0.534 | 1.24e-12 | 0.461 | 1.43e-08 | 0.332 | 8.98e-15 | 0.301 | 1.82e-11 | |
|  | LAG3 | 0.071 | 3.81e-01 | 0.17 | 4.68e-02 | 0.383 | 1.62e-19 | 0.393 | 4.48e-19 | |
|  | TIM-3 | 0.681 | 0e+00 | 0.532 | 2.20e-11 | 0.69 | 3.93e-74 | 0.692 | 1.78e-69 | |
|  | GZMB | 0.478 | 4.14e-10 | 0.344 | 3.80e-05 | 0.191 | 1.31e-05 | 0.211 | 3.37e-06 | |
| Neutrophils | CD66b | -0.077 | 3.47e-01 | -0.175 | 4.12e-02 | 0.053 | 2.31e-01 | 0.047 | 3.04e-01 | |
|  | CD11b | 0.582 | 0e+00 | 0.441 | 6.83e-08 | 0.594 | 0e+00 | 0.59 | 3.84e-46 | |
|  | CCR7 | 0.587 | 0e+00 | 0.538 | 1.21e-11 | 0.348 | 3.82e-16 | 0.348 | 4.43e-15 | |
| Monocyte | CD86 | 0.767 | 0e+00 | 0.666 | 6.22e-19 | 0.721 | 5.79e-84 | 0.720 | 1.55e-79 | |
|  | CD115 | 0.69 | 0e+00 | 0.579 | 1.19e-13 | 0.628 | 0e+00 | 0.623 | 1.10e-52 | |
| M1 Macrophage | INOS | -0.083 | 3.06e-01 | 0.001 | 9.93e-01 | -0.266 | 8.43e-10 | -0.261 | 7.19e-09 | |
|  | IRF5 | 0.471 | 1.19e-09 | 0.262 | 1.97e-03 | 0.63 | 0e+00 | 0.628 | 7.37e-54 | |
|  | COX2 | 0.445 | 1.22e-08 | 0.34 | 4.69e-05 | 0.088 | 4.62e-02 | 0.061 | 1.86e-01 | |
| M2 Macrophage | CD163 | 0.835 | 0e+00 | 0.795 | 4.57e-31 | 0.776 | 0e+00 | 0.775 | 6.32e-97 | |
|  | VSIG4 | 0.857 | 0e+00 | 0.774 | 1.57e-28 | 0.773 | 0e+00 | 0.77 | 5.88e-95 | |
|  | MS4A4A | 1 | 0e+00 | 1 | 0e+00 | 1 | 0e+00 | 1 | 0e+00 | |
| TAM | CCL2 | 0.601 | 0e+00 | 0.475 | 4.54e-09 | 0.442 | 0e+00 | 0.432 | 3.96e-23 | |
|  | CD68 | 0.764 | 0e+00 | 0.653 | 4.92e-18 | 0.81 | 0e+00 | 0.808 | 3.09e-111 | |
|  | IL10 | 0.795 | 1.35e-34 | 0.681 | 5.13e-20 | 0.589 | 1.37e-49 | 0.566 | 8.17e-42 | |
| CAF | FAP | 0.398 | 3.61e-07 | 0.288 | 6.35e-04 | 0.225 | 2.51e-07 | 0.206 | 5.32e-06 | |
| Immune cell | Gene markers | MS4A6A-GBM | | | | MS4A6A-LGG | | | | |
|  |  | none | | purity | | none | | purity | | |
|  |  | cor | p | cor | p | cor | p | cor | p | |
| B cell | CD19 | 0.312 | 8.64e-05 | 0.326 | 1.73e-04 | 0.406 | 7.28e-22 | 0.375 | 2.22e-17 | |
|  | CD79A | 0.351 | 8.66e-06 | 0.405 | 9.31e-07 | 0.406 | 7.33e-22 | 0.42 | 7.42e-22 | |
| T cell (general) | CD3D | 0.691 | 4.87e-23 | 0.582 | 8.7e-14 | 0.539 | 2.95e-40 | 0.543 | 5.34e-38 | |
|  | CD3E | 0.627 | 0e+00 | 0.544 | 6.34e-12 | 0.579 | 1.56e-47 | 0.595 | 3.8e-47 | |
|  | CD2 | 0.694 | 0e+00 | 0.605 | 5.07e-15 | 0.602 | 2.88e-52 | 0.613 | 9.83e-51 | |
| CD8+ T cell | CD8A | 0.335 | 2.52e-05 | 0.243 | 4.18e-03 | 0.165 | 1.72e-04 | 0.163 | 3.49e-04 | |
|  | CD8B | 0.473 | 9.89e-10 | 0.393 | 1.97e-06 | 0.242 | 2.75e-08 | 0.24 | 1.03e-07 | |
| Treg | FOXP3 | 0.473 | 9.89e-10 | 0.121 | 1.58e-01 | -0.154 | 4.66e-04 | 0.292 | 3.46e-08 | |
|  | CCR8 | 0.497 | 6.11e-11 | 0.462 | 1.33e-08 | 0.22 | 4.62e-07 | 0.227 | 5.35e-07 | |
|  | STAT5B | -0.371 | 2.86e-06 | -0.199 | 1.99e-02 | 0.068 | 1.25e-01 | 0.075 | 1.02e-01 | |
|  | TGFB1 | 0.434 | 2.92e-08 | 0.319 | 1.43e-04 | 0.612 | 3.01e-54 | 0.608 | 1.39e-49 | |
| T cell exhaustion | PD-1 | 0.407 | 2.35e-07 | 0.362 | 1.39e-05 | 0.567 | 3.03e-45 | 0.568 | 4e-42 | |
|  | CTLA4 | 0.528 | 2.33e-12 | 0.45 | 3.36e-08 | 0.386 | 9.29e-20 | 0.359 | 5.66e-16 | |
|  | LAG3 | 0.058 | 4.75e-01 | 0.162 | 5.78e-02 | 0.423 | 8.3e-24 | 0.431 | 4.81e-23 | |
|  | TIM-3 | 0.775 | 0e+00 | 0.66 | 1.74e-18 | 0.703 | 3.97e-78 | 0.714 | 1.31e-75 | |
|  | GZMB | 0.504 | 3.2e-11 | 0.373 | 7.26e-06 | 0.318 | 1.29e-13 | 0.342 | 1.4e-14 | |
| Neutrophils | CD66b | -0.137 | 9.19e-02 | -0.228 | 7.31e-03 | 0.024 | 5.86e-01 | 0.011 | 8.04e-01 | |
|  | CD11b | 0.562 | 0e+00 | 0.398 | 1.46e-06 | 0.588 | 2.94e-49 | 0.593 | 9.07e-47 | |
|  | CCR7 | 0.553 | 0e+00 | 0.501 | 4.43e-10 | 0.428 | 2.12e-24 | 0.436 | 1.18e-23 | |
| Monocyte | CD86 | 0.832 | 0e+00 | 0.752 | 3.26e-26 | 0.724 | 8.1e-85 | 0.738 | 1.99e-83 | |
|  | CD115 | 0.708 | 0e+00 | 0.59 | 3.14e-14 | 0.579 | 1.04e-45 | 0.568 | 3.18e-42 | |
| M1 Macrophage | INOS | -0.087 | 2.84e-01 | -0.017 | 8.46e-01 | -0.235 | 9.84e-08 | -0.233 | 2.48e-07 | |
|  | IRF5 | 0.571 | 0e+00 | 0.392 | 2.21e-06 | 0.655 | 1.67e-64 | 0.669 | 2.64e-63 | |
|  | COX2 | 0.315 | 7.85e-05 | 0.152 | 7.65e-02 | 0.099 | 2.45e-02 | 0.076 | 9.62e-02 | |
| M2 Macrophage | CD163 | 0.685 | 0e+00 | 0.591 | 2.80e-14 | 0.758 | 2.83e-97 | 0.75 | 1.35e-87 | |
|  | VSIG4 | 0.829 | 0e+00 | 0.737 | 1.00e-24 | 0.659 | 1.21e-65 | 0.651 | 6.46e-59 | |
|  | MS4A4A | 0.919 | 0e+00 | 0.884 | 2.04e-46 | 0.896 | 7.58e-183 | 0.893 | 1.68e-167 | |
| TAM | CCL2 | 0.577 | 0e+00 | 0.406 | 8.81e-07 | 0.481 | 2.71e-31 | 0.466 | 3.79e-27 | |
|  | CD68 | 0.74 | 0e+00 | 0.623 | 4.23e-16 | 0.803 | 2.13e-117 | 0.805 | 4.33e-110 | |
|  | IL10 | 0.834 | 6.75e-41 | 0.681 | 5.13e-20 | 0.622 | 1.61e-56 | 0.601 | 2.81e-48 | |
| CAF | FAP | 0.382 | 1.09e-06 | 0.255 | 2.63e-03 | 0.235 | 6.85e-08 | 0.221 | 1.08e-06 | |
| Immune cell | Gene markers | MS4A7-GBM | | | | MS4A7-LGG | | | | |
|  |  | none | | purity | | none | | purity | | |
|  |  | cor | p | cor | p | cor | p | cor | p | |
| B cell | CD19 | 0.312 | 8.65e-05 | 0.33 | 8.05e-05 | 0.381 | 3.1e-19 | 0.353 | 1.72e-15 | |
|  | CD79A | 0.346 | 1.19e-05 | 0.365 | 1.16e-05 | 0.428 | 2.13e-24 | 0.446 | 9.98e-25 | |
| T cell (general) | CD3D | 0.582 | 2.91e-15 | 0.418 | 3.66e-07 | 0.414 | 8.99e-23 | 0.398 | 1.24e-19 | |
|  | CD3E | 0.59 | 1.07e-15 | 0.483 | 2.19e-09 | 0.447 | 1.02e-26 | 0.449 | 4.03e-25 | |
|  | CD2 | 0.621 | 1.05e-17 | 0.496 | 7.14e-10 | 0.458 | 4.58e-28 | 0.461 | 1.75e-26 | |
| CD8+ T cell | CD8A | 0.33 | 3.08e-05 | 0.233 | 6.04e-03 | 0.066 | 1.36e-01 | 0.02 | 6.69e-01 | |
|  | CD8B | 0.418 | 7.54e-08 | 0.308 | 2.48e-04 | 0.205 | 2.66e-06 | 0.168 | 2.27e-04 | |
| Treg | FOXP3 | 0.244 | 2.39e-03 | 0.175 | 4.04e-02 | -0.163 | 2.02e-04 | -0.145 | 1.48e-03 | |
|  | CCR8 | 0.449 | 5.93e-09 | 0.385 | 3.42e-06 | 0.178 | 4.58e-05 | 0.181 | 7.1e-05 | |
|  | STAT5B | -0.219 | 6.44e-03 | -0.009 | 9.19e-01 | 0.149 | 6.85e-04 | 0.185 | 4.57e-05 | |
|  | TGFB1 | 0.482 | 2.9e-10 | 0.394 | 1.92e-06 | 0.698 | 1.6e-76 | 0.681 | 1.66e-66 | |
| T cell exhaustion | PD-1 | 0.498 | 5.51e-11 | 0.464 | 1.16e-08 | 0.547 | 1.56e-41 | 0.544 | 3.41e-38 | |
|  | CTLA4 | 0.556 | 8.66e-14 | 0.47 | 6.64e-09 | 0.381 | 3.44e-19 | 0.351 | 2.71e-15 | |
|  | LAG3 | 0.216 | 7.22e-03 | 0.335 | 6.28e-05 | 0.406 | 6.09e-22 | 0.426 | 1.84e-27 | |
|  | TIM-3 | 0.821 | 1.18e-38 | 0.75 | 5.12e-26 | 0.808 | 4.19e-120 | 0.808 | 1.61e-111 | |
|  | GZMB | 0.44 | 1.23e-08 | 0.283 | 8.01e-04 | 0.157 | 3.52e-04 | 0.183 | 5.81e-05 | |
| Neutrophils | CD66b | -0.001 | 9.94e-01 | -0.067 | 4.35e-01 | 0.041 | 3.5e-01 | 0.029 | 5.33e-01 | |
|  | CD11b | 0.635 | 1.12e-18 | 0.514 | 1.37e-10 | 0.698 | 1.01e-76 | 0.687 | 4.79e-68 | |
|  | CCR7 | 0.575 | 7.74e-15 | 0.517 | 1.03e-10 | 0.358 | 5.01e-17 | 0.361 | 3.4e-16 | |
| Monocyte | CD86 | 0.858 | 1.96e-45 | 0.801 | 8.06e-32 | 0.826 | 3.04e-130 | 0.828 | 1.56e-121 | |
|  | CD115 | 0.758 | 7.61e-30 | 0.675 | 1.4e-19 | 0.741 | 6.98e-91 | 0.733 | 8.59e-82 | |
| M1 Macrophage | INOS | -0.115 | 1.56e-01 | -0.038 | 6.6e-01 | -0.247 | 1.36e-08 | -0.252 | 2.45e-08 | |
|  | IRF5 | 0.628 | 3.46e-18 | 0.486 | 1.69e-09 | 0.73 | 4.2e-87 | 0.722 | 3.82e-78 | |
|  | COX2 | 0.326 | 4.03e-05 | 0.177 | 3.84e-02 | 0.145 | 9.9e-04 | 0.119 | 9.22e-03 | |
| M2 Macrophage | CD163 | 0.641 | 4.68e-19 | 0.517 | 9.82e-11 | 0.639 | 1.73e-50 | 0.635 | 2.64e-35 | |
|  | VSIG4 | 0.778 | 3.16e-32 | 0.661 | 1.44e-18 | 0.789 | 6.55e-111 | 0.774 | 2.51e-96 | |
|  | MS4A4A | 0.863 | 1.52e-46 | 0.792 | 9.06e-21 | 0.888 | 7.31e-176 | 0.882 | 2.4e-187 | |
| TAM | CCL2 | 0.438 | 1.51e-08 | 0.219 | 1.01e-02 | 0.487 | 4.87e-32 | 0.469 | 1.5e-27 | |
|  | CD68 | 0.815 | 1.17e-37 | 0.744 | 2.06e-25 | 0.863 | 2.78e-154 | 0.855 | 1.01e-137 | |
|  | IL10 | 0.777 | 3.63e-32 | 0.663 | 1.03e-18 | 0.608 | 1.9e-53 | 0.577 | 7.33e-44 | |
| CAF | FAP | 0.342 | 1.51e-05 | 0.193 | 2.35e-02 | 0.22 | 4.27e-07 | 0.183 | 5.83e-05 | |
| Immune cell | Gene markers | TMEM176A-GBM | | | | TMEM176A-LGG | | | | |
|  |  | none | | purity | | none | | purity | | |
|  |  | cor | p | cor | p | cor | p | cor | p | |
| B cell | CD19 | 0.169 | 3.69e-02 | 0.187 | 2.88e-02 | 0.289 | 2.26e-11 | 0.228 | 4.61e-07 | |
|  | CD79A | 0.231 | 4.07e-03 | 0.25 | 3.28e-03 | 0.109 | 1.36e-02 | 0.109 | 1.69e-02 | |
| T cell (general) | CD3D | 0.343 | 1.43e-05 | 0.245 | 3.84e-03 | 0.454 | 1.41e-27 | 0.392 | 4.98e-19 | |
|  | CD3E | 0.258 | 1.32e-03 | 0.163 | 5.76e-02 | 0.458 | 4.22e-28 | 0.413 | 4.28e-21 | |
|  | CD2 | 0.312 | 9.52e-05 | 0.213 | 1.23e-02 | 0.45 | 4.33e-27 | 0.345 | 7.92e-15 | |
| CD8+ T cell | CD8A | 0.241 | 2.76e-03 | 0.165 | 5.39e-02 | 0.374 | 1.42e-18 | 0.272 | 1.44e-09 | |
|  | CD8B | 0.291 | 2.73e-04 | 0.225 | 8.09e-03 | 0.347 | 4.85e-16 | 0.257 | 1.22e-08 | |
| Treg | FOXP3 | 0.062 | 4.44e-01 | 0.042 | 6.26e-01 | -0.022 | 6.14e-01 | -0.013 | 7.83e-01 | |
|  | CCR8 | 0.199 | 1.36e-02 | 0.139 | 1.06e-01 | 0.171 | 9.8e-05 | 0.17 | 1.89e-04 | |
|  | STAT5B | -0.086 | 2.91e-01 | 0.046 | 5.92e-01 | -0.308 | 8.36e-13 | -0.234 | 2.23e-07 | |
|  | TGFB1 | 0.219 | 6.53e-03 | 0.175 | 4.04e-02 | 0.435 | 0e+00 | 0.371 | 4.35e-17 | |
| T cell exhaustion | PD-1 | 0.143 | 7.87e-02 | 0.09 | 2.98e-01 | 0.394 | 1.18e-20 | 0.358 | 6.98e-16 | |
|  | CTLA4 | 0.179 | 2.68e-02 | 0.104 | 2.28e-01 | 0.272 | 3.51e-10 | 0.214 | 2.31e-06 | |
|  | LAG3 | -0.067 | 4.1e-01 | -0.082 | 3.43e-01 | 0.024 | 5.84e-01 | 0.064 | 1.60e-01 | |
|  | TIM-3 | 0.283 | 4.19e-04 | 0.143 | 9.61e-02 | 0.526 | 4.65e-38 | 0.444 | 1.70e-24 | |
|  | GZMB | 0.256 | 1.38e-03 | 0.197 | 2.08e-02 | 0.166 | 1.52e-04 | 0.178 | 8.78e-05 | |
| Neutrophils | CD66b | -0.061 | 4.57e-01 | -0.092 | 2.83e-01 | 0.055 | 2.1e-01 | 0.051 | 2.62e-01 | |
|  | CD11b | 0.239 | 2.98e-03 | 0.121 | 158e-01 | 0.466 | 0e+00 | 0.359 | 5.12e-16 | |
|  | CCR7 | 0.261 | 1.14e-03 | 0.219 | 1.01e-02 | 0.318 | 1.49e-13 | 0.294 | 5.82e-11 | |
| Monocyte | CD86 | 0.286 | 3.66e-04 | 0.162 | 5.85e-02 | 0.49 | 1.67e-32 | 0.398 | 1.35e-19 | |
|  | CD115 | 0.298 | 1.98e-04 | 0.191 | 2.51e-02 | 0.382 | 0e+00 | 0.257 | 1.15e-08 | |
| M1 Macrophage | INOS | 0.154 | 5.8e-02 | 0.205 | 1.60e-02 | 0.002 | 9.63e-01 | -0.024 | 5.97e-01 | |
|  | IRF5 | 0.239 | 2.94e-03 | 0.12 | 1.64e-01 | 0.493 | 0e+00 | 0.409 | 1.09e-20 | |
|  | COX2 | 0.258 | 1.34e-03 | 0.213 | 1.23e-02 | 0.091 | 3.95e-02 | 0.011 | 8.04e-01 | |
| M2 Macrophage | CD163 | 0.326 | 4.36e-05 | 0.271 | 1.33e-03 | 0.183 | 2.98e-05 | 0.169 | 2.08e-04 | |
|  | VSIG4 | 0.322 | 5.41e-05 | 0.217 | 1.09e-02 | 0.324 | 5.58e-14 | 0.235 | 1.92e-07 | |
|  | MS4A4A | 0.4 | 3.76e-07 | 0.342 | 4.35e-05 | 0.285 | 5.11e-11 | 0.267 | 3.11e-09 | |
| TAM | CCL2 | 0.401 | 3.65e-07 | 0.314 | 1.90e-04 | 0.404 | 0e+00 | 0.349 | 3.53e-15 | |
|  | CD68 | 0.279 | 4.98e-04 | 0.163 | 5.64e-02 | 0.465 | 0e+00 | 0.404 | 3.25e-20 | |
|  | IL10 | 0.406 | 1.97e-07 | 0.326 | 1.03e-04 | 0.394 | 1.34e-20 | 0.345 | 7.92e-15 | |
| CAF | FAP | 0.242 | 2.6e-03 | 0.174 | 4.14e-02 | 0.371 | 3.07e-18 | 0.274 | 1.19e-09 | |
| Immune cell | Gene markers | TMEM176B-GBM | | | | TMEM176B-LGG | | | |  |
|  |  | none | | purity | | none | | purity | |  |
|  |  | cor | p | cor | p | cor | p | cor | p |  |
| B cell | CD19 | 0.206 | 1.06e-02 | 0.226 | 7.88e-03 | 0.331 | 1.28e-14 | 69 | 2.32e-09 | |
|  | CD79A | 0.272 | 6.66e-04 | 0.282 | 8.39e-04 | 0.171 | 9.26e-05 | 0.179 | 7.96e-05 | |
| T cell (general) | CD3D | 0.391 | 5.85e-07 | 0.278 | 1.01e-03 | 0.52 | 4.92e-37 | 0.469 | 1.84e-27 | |
|  | CD3E | 0.306 | 1.2e-04 | 0.195 | 2.25e-02 | 0.532 | 4.86e-39 | 0.502 | 1.47e-31 | |
|  | CD2 | 0.358 | 5.64e-06 | 0.243 | 4.28e-03 | 0.533 | 3.18e-39 | 0.502 | 7.32e-32 | |
| CD8+ T cell | CD8A | 0.251 | 1.76e-03 | 0.163 | 5.72e-02 | 0.392 | 0e+00 | 0.302 | 1.56e-11 | |
|  | CD8B | 0.305 | 1.35-04 | 0.217 | 1.1e-02 | 0.357 | 5.6e-17 | 0.278 | 6.49e-10 | |
| Treg | FOXP3 | 0.098 | 2.27e-01 | 0.078 | 3.65e-01 | -0.051 | 2.48e-01 | -0.039 | 3.98e-01 | |
|  | CCR8 | 0.274 | 6.06e-04 | 0.216 | 1.11e-02 | 0.188 | 1.7e-05 | 0.189 | 3.25e-05 | |
|  | STAT5B | -0.108 | 1.83e-01 | 0.044 | 6.10e-01 | -0.269 | 5.59e-10 | -0.198 | 1.29e-05 | |
|  | TGFB1 | 0.279 | 5.12e-04 | 0.224 | 8.41e-03 | 0.498 | 0e+00 | 0.446 | 1.02e-24 | |
| T cell exhaustion | PD-1 | 0.157 | 5.32e-02 | 0.086 | 3.17e-01 | 0.449 | 6.01e-27 | 0.42 | 7.41e-22 | |
|  | CTLA4 | 0.218 | 6.91e-03 | 0.131 | 1.27e-01 | 0.343 | 1.2e-15 | 0.293 | 6.14e-11 | |
|  | LAG3 | -0.075 | 3.55e-01 | -0.085 | 3.22e-01 | 0.066 | 1.35e-01 | 0.106 | 2.04e-02 | |
|  | TIM-3 | 0.313 | 8.74e-05 | 0.157 | 6.72e-02 | 0.604 | 1.07e-52 | 0.539 | 1.98e-37 | |
|  | GZMB | 0.297 | 1.93e-04 | 0.222 | 9.21e-03 | 0.229 | 1.42e-07 | 0.249 | 3.48e-08 | |
| Neutrophils | CD66b | -0.05 | 5.4e-01 | -0.071 | 4.11e-01 | 0.048 | 2.8e-01 | 0.043 | 3.44e-01 | |
|  | CD11b | 0.282 | 4.29e-04 | 0.154 | 7.16e-02 | 0.546 | 0e+00 | 0.461 | 1.61e-26 | |
|  | CCR7 | 0.306 | 1.3e-04 | 0.263 | 1.94e-03 | 0.36 | 3e-17 | 0.343 | 1.32e-14 | |
| Monocyte | CD86 | 0.334 | 2.75e-05 | 0.195 | 2.28e-02 | 0.578 | 2.93e-47 | 0.507 | 1.45e-32 | |
|  | CD115 | 0.353 | 8.87e-06 | 0.236 | 5.54e-03 | 0.466 | 0e+00 | 0.363 | 2.24e-16 | |
| M1 Macrophage | INOS | 0.17 | 3.54e-02 | 0.222 | 9.01e-03 | -0.04 | 3.6e-01 | -0.06 | 1.91e-01 | |
|  | IRF5 | 0.266 | 9.05e-04 | 0.127 | 1.40e-01 | 0.567 | 0e+00 | 0.502 | 7.94e-32 | |
|  | COX2 | 0.279 | 4.98e-04 | 0.227 | 7.77e-03 | 0.109 | 1.29e-02 | 0.033 | 4.70e-01 | |
| M2 Macrophage | CD163 | 0.393 | 6.22e-07 | 0.342 | 4.30e-05 | 0.296 | 8.57e-12 | 0.289 | 1.27e-10 | |
|  | VSIG4 | 0.386 | 1e-06 | 0.283 | 8.09e-04 | 0.409 | 0e+00 | 0.332 | 9.09e-14 | |
|  | MS4A4A | 0.463 | 2.58e-09 | 0.401 | 1.19e-06 | 0.392 | 0e+00 | 0.381 | 5.43e-18 | |
| TAM | CCL2 | 0.436 | 2.55e-08 | 0.336 | 6.10e-05 | 0.47 | 0e+00 | 0.425 | 2.47e-22 | |
|  | CD68 | 0.328 | 3.89e-05 | 0.207 | 1.54e-02 | 0.567 | 0e+00 | 0.514 | 1.36e-33 | |
|  | IL10 | 0.458 | 2.65e-09 | 0.37 | 8.73e-06 | 0.498 | 1.59e-32 | 0.452 | 2.07e-25 | |
| CAF | FAP | 0.301 | 1,57e-04 | 0.232 | 6.3e-03 | 0.385 | 1.09e-19 | 0.295 | 4.99e-11 | |
